# Modelling the transport of fluid through heterogeneous, whole tumours *in silico*

**DOI:** 10.1371/journal.pcbi.1006751

**Published:** 2019-06-21

**Authors:** Paul W. Sweeney, Angela d’Esposito, Simon Walker-Samuel, Rebecca J. Shipley

**Affiliations:** 1 Mechanical Engineering, University College London, London, United Kingdom; 2 Centre for Advanced Biomedical Engineering, University College London, London, United Kingdom; University of Pennsylvania, UNITED STATES

## Abstract

Cancers exhibit spatially heterogeneous, unique vascular architectures across individual samples, cell-lines and patients. This inherently disorganised collection of leaky blood vessels contribute significantly to suboptimal treatment efficacy. Preclinical tools are urgently required which incorporate the inherent variability and heterogeneity of tumours to optimise and engineer anti-cancer therapies. In this study, we present a novel computational framework which incorporates whole, realistic tumours extracted *ex vivo* to efficiently simulate vascular blood flow and interstitial fluid transport *in silico* for validation against *in vivo* biomedical imaging. Our model couples Poiseuille and Darcy descriptions of vascular and interstitial flow, respectively, and incorporates spatially heterogeneous blood vessel lumen and interstitial permeabilities to generate accurate predictions of tumour fluid dynamics. Our platform enables highly-controlled experiments to be performed which provide insight into how tumour vascular heterogeneity contributes to tumour fluid transport. We detail the application of our framework to an orthotopic murine glioma (GL261) and a human colorectal carcinoma (LS147T), and perform sensitivity analysis to gain an understanding of the key biological mechanisms which determine tumour fluid transport. Finally we mimic vascular normalization by modifying parameters, such as vascular and interstitial permeabilities, and show that incorporating realistic vasculatures is key to modelling the contrasting fluid dynamic response between tumour samples. Contrary to literature, we show that reducing tumour interstitial fluid pressure is not essential to increase interstitial perfusion and that therapies should seek to develop an interstitial fluid pressure gradient. We also hypothesise that stabilising vessel diameters and permeabilities are not key responses following vascular normalization and that therapy may alter interstitial hydraulic conductivity. Consequently, we suggest that normalizing the interstitial microenvironment may provide a more effective means to increase interstitial perfusion within tumours.

## Author summary

The structure of tumours varies widely, with dense and chaotically-formed networks of blood vessels that differ between each individual tumour and even between different regions of the same tumour. This atypical environment can inhibit the delivery of anti-cancer therapies. Computational tools are urgently required which facilitate a deeper understanding of the relationship between blood vessel architectures and therapeutic response. We have developed a computational framework which integrates the complex tumour vascular architecture to predict fluid transport across all lengths scales in whole tumours. We apply our model to two tumour cell-lines and show that differences in their inherent vascular structures influence flow through cancerous tissue. We also use our platform to predict the fluid dynamic response following vascular normalization therapy in realistic, static tumour networks and show that the response is dependent on tumour vascular architecture. We hypothesise that therapy may alter the permeability of interstitial tissue to fluid transport and show that lowering interstitial fluid pressure is not a necessary therapeutic outcome to increase tumour perfusion.

## Introduction

Architectural heterogeneities in cancerous tissue limit the delivery of anti-cancer drugs by inhibiting their ability to circumnavigate the entire tumour to all cancerous cells [1]. In solid tumours, drug penetration to the tumour core is hindered by physiological barriers which can limit the delivery of targeted agents, with penetration exacerbated by the size of the agent [1–5]. Consequently, preclinical tools which provide a better understanding of therapy interactions within the tumour microenvironment are urgently required in order to increase treatment efficacy. *In silico* modelling is one such tool which can meet this need by testing novel therapeutic strategies at a much faster rate and cheaper cost than preclinical experimentation [6].

For a systemically-administered agent to effectively target diseased tissue, it must travel from the site of delivery to the site of disease, whilst minimally interacting with normal tissues and not degrading [7]. This is difficult to achieve in tumours since atypical endothelial proliferation of tumour vasculature leads to spatial variations in vascular density and branching patterns, distorted and enlarged vessels, and a highly convoluted network topology [8–10]. Further, vascular permeability is heightened and heterogeneous and so these immature blood vessels are generally leakier than those in normal tissue [3, 11].

The irregular microenivronment is typically characterised by hypoxia, acidosis and elevated interstitial fluid pressure (IFP) [12–14], which drive both tumour vascular proliferation and resistance to therapy [15]. Here, drug delivery may be hindered by the atypical nature of the tumour interstitium. The extracellular matrix (ECM) consists of a cross-linked dense network of collagen and elastin fibres, far denser than usually seen in normal tissue [16]. A denser matrix can inhibit oxygen and nutrient delivery, as well as providing significant resistance to the advection and diffusion of therapeutic particles [1], since key determinants of intratumoural fluid and mass delivery include tissue hydraulic conductivity and vascular compliance [17]. Several therapeutic interventions have sought to limit the effects of these physical barriers by manipulating the microenvironment to enhance the delivery of macromolecular agents [16, 18]. For example, normalising the tumour vasculature to reduce vessel permeability thereby increasing drug penetration [12]; and manipulating the connective tissue, and therefore interstitial hydraulic conductivity, using a platelet-derived growth factor (PDGF) antagonist to reduce tumour IFP [19].

Heterogeneities in the underlying morphology of tumours, such as vessel diameters and lengths, and inter-branch distance, exist across individual tumours and tumour cell-lines [20]. These variations in tumour architecture lead to spatial variability in drug efficacy, which complicate efforts to design effective treatment strategies [7]. Experimental efforts have been made to understand the effects of tumour heterogeneity on fluid interactions across tumours, for example, wick-in-needle has been used to measure IFP across tumours [21–23]. However, this method disturbs the local microenvironment and only provides an IFP measurement at individual locations. Non-invasive methods have also been developed to estimate tumour IFP [24, 25]. For example, convection-MRI, which measures low-velocity flow in tumours at a resolution of ∼ 250 *μ*m *in vivo* [26]. However, these methods fail to capture full spatial maps of flow at the micron-scale which are crucial to understanding how the combined intra- and extravascular spatial flow heterogeneities occurring at the scale of blood vessels affects the macro-scale flow dynamics and consequent delivery of drugs within a solid tumour. Biomedical imaging complemented by *in silico* methods provides scope to provide such detail.

Mathematical models have been used to investigate the tumour microenvironment and have provided detailed insights which may otherwise be unavailable experimentally. Seminal models have indicated that a leaky tumour vasculature induces elevated IFP, reduced fluid penetration into the interstitium [14, 27], and a non-uniform distribution of drug delivered to solid tumours [2, 3, 11]. Further, they have defined conventional IFP profiles in tumours—a uniform pressure at the core, with a large decreasing gradient towards the periphery. However, these models generally average spatially over the tumour vasculature and so fail to capture the micron-scale flow dynamics; and they assign a fixed pressure boundary condition on the periphery of the tumour which may artificial induce these conventional IFP profiles.

Subsequent studies have sought to incorporate the spatially heterogeneous effects of tumour vasculature using computer-generated synthetic networks which retain key features of tumour vascular architecture [28–34], or by integrating spatial variations in vascular permeability parametrised against *in vivo* experimentation [35, 36]. More recently, a hybrid image-based framework has been developed which combines realistic tumour vascular architectures and *in silico* modelling to predict tumour vascular blood flow [37] and intravascular tumour oxygenation [38]. However, computational models are urgently required which incorporate these highly detailed data to enable predictions of fluid and mass transport to the surrounding tissue [6].

Recent advances in *ex vivo* optical imaging of cleared tissue specimens have enabled large samples (up to 2 cm^3^ with > 10^5^ blood vessels) to be imaged in three-dimensions, at resolutions down to a few microns [39]. We have developed a platform called REANIMATE (REAlistic Numerical Image-based Modelling of biologicAL Tissue substratEs) which combines optical imaging of cleared tissue with mathematical modelling and *in vivo* imaging, within a unified framework, to generate quantitative, testable predictions regarding tumour transport [40]. The platform uses high-resolution imaging data from large, intact, optically-cleared tissue samples to make *in silico* predictions of blood flow, vascular exchange and interstitial transport. REANIMATE enables new hypotheses to be generated and tested in a manner that would be challenging or impossible in a conventional experimental setting. We have previously used REANIMATE to explore the impact of vascular network topology on fluid transport and vascular disrupting therapy (Oxi4503) to two colorectal cell-lines (LS147T and SW1222) [40].

We develop here a computational model to efficiently simulate both intra- and extravascular fluid transport across large, discrete microvascular networks [40]. Our model simulates Poiseuille flow through the vasculature using the optimisation scheme of Fry et al. [41], parametrised and validated against *in vivo* ASL-MRI data [40]. Following a similar Green’s function method for oxygen transport [42], the vascular component is coupled, via a discrete set of point sources of flux, to a Darcy model which simulates the effective fluid transport in the porous interstitium. A linear system is formed whereby only the source strengths need to be resolved, making it more computationally efficient compared to finite difference or element methods which require a spatial, numerically-discretised mesh [42].

In this study, we detail the generalised model which allows for spatially heterogeneous hydraulic conductances and conductivities. A comprehensive description is provided of its application to whole tumour vascular networks. We then apply our model to an orthotopic murine glioma (GL261) and a human colorectal carcinoma xenograft (LS147T) and reproduce physiological conditions observed in literature. We perform sensitivity analysis to the model parameters associated with transvascular fluid delivery, such as vascular hydraulic conductance and interstitial hydraulic conductivity, to explore the impact on the tumour IFP and interstitial fluid velocity (IFV) profiles.

Finally, we use our computational framework to explore the biomechanics underpinning vascular normalization. Vascular normalization is a method that applies anti-angiogenic therapy to restore tumour vascular structure and function to physiological levels [9, 43–45]. By modifying vascular architecture, therapy aims to normalize tumour perfusion and oxygenation, thereby increasing the efficacy of chemo, radio and immunotherapy [44, 46–48].

Preclinical and clinical evidence indicates that anti-VEGF (vascular endothelial growth factor) therapy creates a transient window of vessel normalization which improves tumour oxygenation and the delivery of therapeutic agents [15, 49]. However, the extent and window of normalization, and the duration and dosage of an anti-cancer drug varies with tumour type [18]. Further, the gold standard for detecting normalization of tumour blood vessels (such as perfusion, microvessel density, morphology and permeability) in the clinic is via histological staining [50].

Here, we recruit our *in silico* model in combination with realistic, static vasculatures to replicate fluid dynamics changes observed experimentally during vascular normalization [51–53] by modifying parameters such as blood vessel diameters, and vascular and interstitial permeabilities. In doing so we hypothesise which of these biomechanics are responsible for experimental observations following therapy, in addition to how tumour type, and the inherent differences in vascular networks structures, affects tumour IFP and perfusion following normalization therapy.

## Materials and methods

### Acquisition and processing of real-world tumour datasets

Orthotopic murine gliomas and human colorectal carcinoma xenograft from the GL261 and LS147T cell-lines (n = 6 for each), respectively, were grown subcutaneously in 8–10 week old, female mice. Following 10 to 14 days of growth, *in vivo* arterial spin-labelling MRI (ASL-MRI) was performed on a subset of GL261 and LS147T tumours, from which a mean tumour perfusion of 130 ± 50 and 19 ± 8 ml/min/100g was measured [40], respectively. Following perfuse-fixation, tumours were harvested, optically cleared and imaged using optical projection tomography (OPT, Bioptonics, MRC Technologies, Edinburgh). All experiments were performed in accordance with the UK Home Office Animals Scientific Procedures Act 1986 and UK National Cancer Research Institute (NCRI) guidelines [54]. Full details of the experimental protocol is provided in d’Esposito et al. [40].

**Fig 1:**
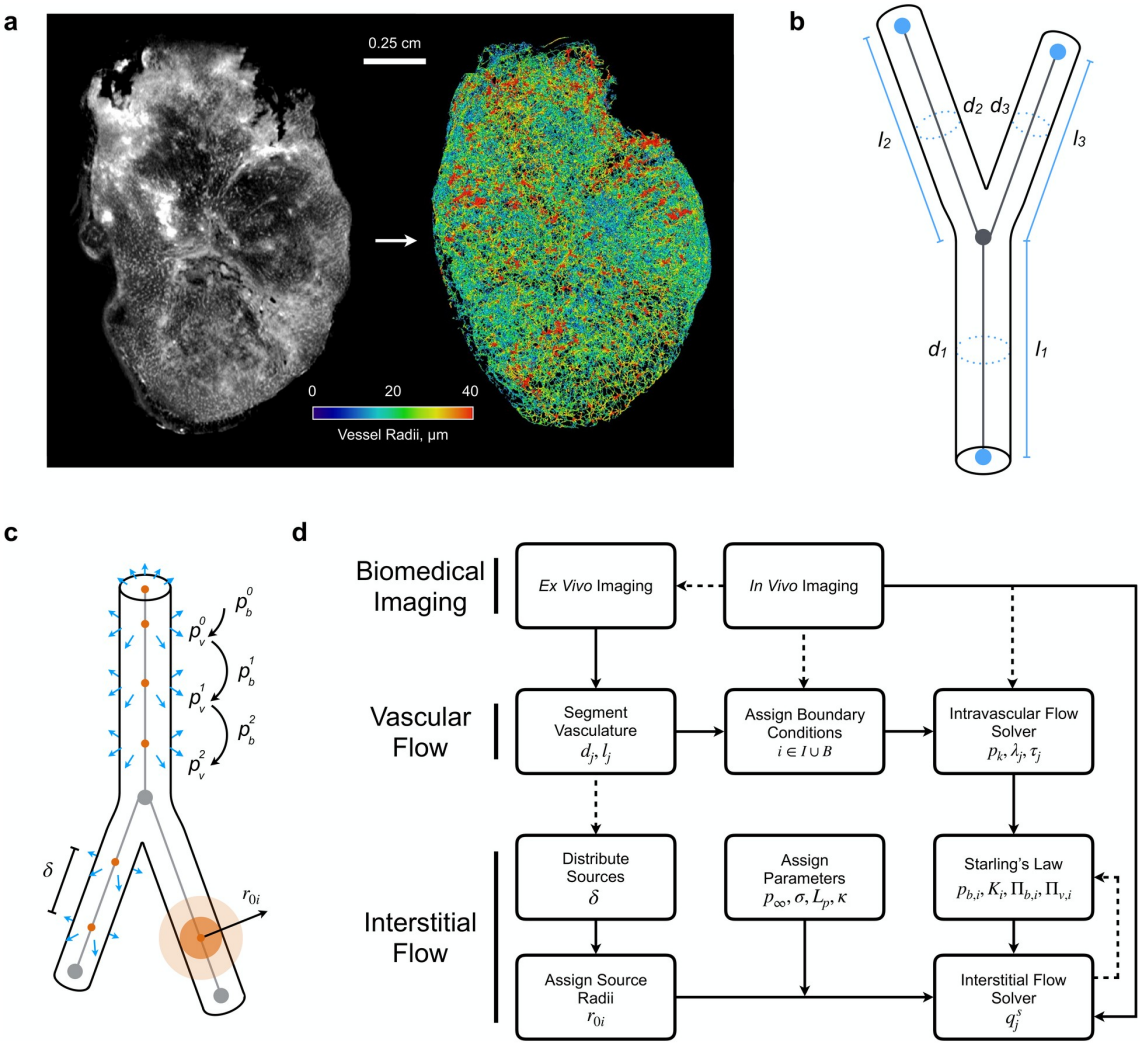
(a) An example of an SW1222 tumour vascular network enhanced using Frangi filters and extracted from the tumour image stack generated by OPT. (b) The skeletonised vasculature is then segmented into a series of interconnected nodes and vessel segments with known diameters, *d*_*i*_ and lengths, *l*_*i*_, for *i* = 1, 2, 3 shown here. (c) A schematic of sensitivity analysis performed on the source parameters: 1) updating intravascular pressure *p*_*i*,*b*_ for *k* iterations where *p*_0,*b*_ is the initial network pressure distribution approximated by the flow estimation algorithm; 2) the spacing *δ* between sources distribution across a branching vessel; and 3) the size of the source radius, *r*_0_. (d) A flow diagram of the computational framework. *In vivo* imaging is performed on vascularised tissue to obtain perfusion data (and literature values of vascular pressures when available) which are used to parameterise and validate the framework. *Ex vivo* imaging is performed on equivalent tissue samples to obtain data on the vascular architecture, including coordinates, vessel diameters and lengths, which are then used to parameterise the vascular flow model. Boundary conditions are assigned (see Fig 2a and 2b) and network intravascular blood pressure is solved. Sources of fluid flux are distributed across the vasculature and assigned a radius equal to their corresponding vessel radius. Interstitial flow parameters are assigned and the model is coupled to the vascular flow compartment via Starling’s Law. Solved source strengths are used to update Starling’s law (*i* ∈ *N*_*s*_). This iterative scheme is terminated once predefined tolerances are reached.

Whole-tumour blood vessel networks were segmented from the OPT data for both tumour types. A combination of three-dimensional Gaussian and Frangi filters were applied to the data to enhance vessel-like structures allowing for the segmentation of the blood vessels from the background (see Fig 1a). Skeletonisation of these thresholded data was performed in Amira (Thermo Fisher Scientific, Hillsboro, OR), which also converted the data into graph format (interconnected network of nodes and segments with associated radii, see Fig 1b). To ensure that vessel structures were accurately represented, three-dimensional networks were visually inspected against two-dimensional imaging slices for an accurate representation of vessel location and thickness. Full details of the validation can be found in the Supplementary Material of d’Esposito et al. [40].

In this study a GL261 and a LS147T tumour network were chosen from the d’Esposito et al. [40] datasets for *in silico* development and testing. Vessel diameters ranged from 17.9 ± 9.3 and 8.9 ± 2.8 *μ*m, with branching lengths of 68.7 ± 48.3 and 88.8 ± 49.4 *μ*m, respectively (see Table 1). Vessel branching angles, inter-vessel distance, radii and tortuosity measures were consistent with data from previous studies that extracted vascular architectures using different methods [20, 40].

**Table 1:** Tumour vascular network statistics.

	GL261	LS147T	Units
No. of Segments	121,212	76,378	-
No. of Nodes	110,062	60,177	-
No. of Boundary Nodes	8,660	15,783	-
Mean Vessel Diameter	17.9 ± 9.3	8.9 ± 2.8	*μ*m
Mean Branching Vessel Length	68.7 ± 48.3	88.8 ± 49.4	*μ*m
Tissue Dimensions	4.3 × 4.1 × 4.6	6.9 × 5.2 × 8.2	mm^3^
Vascular Density	5.07	0.37	%

Segmented network data on the segmented murine orthotopic glioma, GL261, and the human colorectal xenograft, LS147T, vasculature. See Fig 3 for visualisations of both networks.

### Computational model

Our computational framework is compartmentalised into two models. The first predicts blood flow through the tumour vasculature and the second predicts interstitial fluid flow throughout the cancerous tissue through use of non-singular Green’s functions. Our method enables application to whole, large vascular networks (> 2 cm^3^ with > 10^5^ blood vessels), thereby permitting predictions of whole tumour fluid dynamics which incorporate the inherent architectural heterogeneities occurring at the micron-scale.

The intravascular component incorporates the model of Pries et al. [55] to simulate vascular blood flow, where the structural properties of the segmented tumour networks and haemodynamic parameters are used as inputs. Flow or pressure boundary conditions at all terminal nodes in the vascular network are required to predict blood flow throughout the network. These boundary data are very challenging to measure *in vivo*, so we deploy the flow estimation algorithm of Fry et al. [41] to estimate boundary data based on the assumption that the microcirculation is regulated in response to haemodynamics stimuli relating to flow and shear stresses [56]. The scheme estimates unknown boundary conditions by minimising the squared deviation from specified target network wall shear stresses and pressures derived from independent information about typical network haemodynamic properties. In essence, the algorithm removes the need to define conditions at all boundary nodes, to one where simulation sensitivity is weighted towards the definition of these two target parameters. This enables physiologically realistic blood pressure and flow distributions to be estimated across an entire vascular network and has been applied to breast tumour [37], colorectal tumours [40], cortex [57], glioma [40] and skeletal muscle [58].

The second component to our computational model describes fluid transport through the porous interstitium using a Darcy model. Here, the vascular flow solution is coupled to Darcy flow via Starling’s law which describes fluid transport across the endothelium. The vasculature is represented by a discrete set of points sources of flux where the source strengths are defined by the vascular blood flow solution. A similar approach has been applied to simulate O2 transport across various tissues [42, 57, 59] and to predict capillary flow in the absence of these network structures [60]. Our generalised approach enables us to explore the affect of vascular architecture heterogeneity on fluid transport within the interstitium for large-scale vascular networks with spatially heterogeneous tissue and vessel wall permeabilities.

The following sections present the mathematics behind our model, followed by a description detailing its application to large tumour networks.

#### Vascular blood flow

The segmented tumour networks consist of a series of vessel segments connected by nodal junctions or, in the case of boundary nodes, one-segment nodes which form a boundary to the microvascular network (see Fig 1b). We define a positive flow direction from the start node to end node of each vessel segment. Under the assumption of Poiseuille flow and conserving flow at blood vessel junctions, the relationship between nodal pressures, *p*_*k*_ and the boundary boundary fluxes *Q*_0*i*_ is given by

$$\begin{array}{c} \sum_{k\in N}K_{ik}p_{k}=-Q_{0i}\;\text{for}\;i\in I\cup B, \end{array}$$ (1)

where *N* is the set of all nodes, *I* is the set of all interior nodes and *B* is the set of all boundary nodes with known boundary conditions. For all interior nodes, conservation of flux at vessel junctions dictates that *Q*_0*i*_ = 0, however, if *i* is a known boundary node, *Q*_0*i*_ is the inflow (or outflow if negative).

Following the notation of Fry et al. [41], the matrix *K*_*ik*_ represents network conductance

$$\begin{array}{c} K_{ik}=\sum_{j\in S}L_{ij}M_{jk}, \end{array}$$ (2)

where *S* is the set of all segments,

$$\begin{array}{c} L_{ij}=\{\begin{array}{cc} -1,\; & \text{if}\;i\;\text{is}\;\text{the}\;\text{start}\;\text{node}\;\text{of}\;\text{segment}\;j\text{,}\\ +1,\; & \text{if}\;i\;\text{is}\;\text{the}\;\text{end}\;\text{node}\;\text{of}\;\text{segment}\;j\text{,}\\ 0,\; & \text{otherwise,} \end{array} \end{array}$$ (3)

and

$$\begin{array}{c} M_{jk}=\{\begin{array}{cc} +\pi d_{j}^{4}/(128\mu_{j}l_{j}),\; & \text{if}\;k\;\text{is}\;\text{the}\;\text{start}\;\text{node}\;\text{of}\;\text{segment}\;j\text{,}\\ -\pi d_{j}^{4}/(128\mu_{j}l_{j}),\; & \text{if}\;k\;\text{is}\;\text{the}\;\text{end}\;\text{node}\;\text{of}\;\text{segment}\;j\text{,}\\ 0,\; & \text{otherwise,} \end{array} \end{array}$$ (4)

is the matrix of vessel conductances where *l*_*j*_, *d*_*j*_ and *μ*_*j*_ denote the length, diameter and effective blood viscosity of segment *j*, respectively.

We apply empirical *in vivo* blood viscosity laws, which prescribe the effective blood viscosity as a function of vessel diameter and haematocrit, to compute *μ*_*j*_ and consequently incorporate non-Newtonian effects in each individual microvessel [61]. Network haematocrit heterogeneity plays an important part in network flow resistance. However, in this study, we set network haematocrit to 0.45 as we do not have sufficient data to parameterise a haematocrit model at this scale. With future availability of appropriate data, the model has the flexibility to incorporate haematocrit heterogeneity [62].

In the absence of measured flow and pressure data at network boundaries, further assumptions are required to obtain a unique solution. The method proposed by Fry et al. [41] sought to solve a constrained optimisation problem, formulated in terms of a Lagrangian objective function, defined by

$$\begin{array}{l} L=\frac{1}{2}k_{p}\sum_{k\in N}w_{k}{\left(p_{k}-p_{0k}\right)}^{2}+\frac{1}{2}k_{\tau }\sum_{j\in S}\ell_{j}{\left(\tau_{j}-\tau_{0j}\right)}^{2}\\ \;+\sum_{i\in I\cup B}\lambda_{i}\left(\sum_{k\in N}K_{ik}p_{k}+Q_{0i}\right). \end{array}$$ (5)

Here, *p*_0*k*_ is the target pressure at node *k*, *τ*_*j*_ is the wall shear stress in segment *j*, *τ*_0*j*_ is the corresponding target shear stress, *k*_*p*_ and *k*_*τ*_ are weighting factors associated with the pressure and shear deviations from the target values, λ_*i*_ is the Lagrange multiplier associated with node *i* and *w*_*k*_ is the vessel length associated with node *k*. Setting *dL*/*dp*_*i*_ = 0 and combing with (1) yields a sparse linear system with unknowns *p*_*k*_ and λ_*i*_. Assigning a pressure drop to a proportion of boundary nodes forms a well-posed system which can be solved using standard methods [41].

The blood flow estimation model by Fry et al. [41] has been thoroughly tested using mesenteric networks [63] in which blood flow measurements were taken in individual vessels and used to inform parameter estimation [41, 55, 61, 64].

#### Interstitial fluid transport

Darcy’s law has been effectively used to describe the passage of fluids [3, 14, 30–33, 65] or solutes [42, 57] through tissues. In this study, we use Darcy’s law to describe the relationship between the IFP, *p*, and volume-averaged interstitial fluid velocity (IFV), **u**, within the porous interstitial domain:

$$\begin{array}{c} u=-\kappa \nabla p, \end{array}$$ (6)

where *κ* is the hydraulic conductivity of the interstitial tissue. Here we assume that interstitial pressure tends towards a constant value, *p*_∞_, in the far-field region,

$$\begin{array}{c} p\to p_{\infty }\;\text{as}\;\left|x\right|\to \infty . \end{array}$$ (7)

Tumours are leaky due to large pores along a vessel’s lumen, and so the vasculature exhibits a strong fluid and oncotic interaction. Following the approach of Baxter and Jain [3] and subsequent studies [43, 66], we used Starling’s law to describe fluid transport across the endothelium:

$$\begin{array}{c} J_{v}=L_{p}S(\Delta p-\sigma \Delta \Pi ), \end{array}$$ (8)

where *J*_*v*_ and *L*_*p*_ are the fluid flux across and the hydraulic conductance of the vessel wall, respectively, *S* is the surface area of the vasculature, *σ* is the oncotic reflection coefficient and, Δ*p* and ΔΠ are the fluid and oncotic pressure gradients between the vasculature and surrounding tissue.

The tumour vascular architecture is used to spatially parameterise the locations of a discrete set of sources of flux into the interstitial domain. Assuming these sources both supply or drain the interstitium, conservation of mass yields

$$\begin{array}{c} \nabla \cdot u=-\kappa \nabla^{2}p=\sum_{j}\phi_{j}\left(x\right)\delta \left(x-x_{j}\right), \end{array}$$ (9)

where **x**_*j*_ and *ϕ*_*j*_ are the spatial coordinates and strength, respectively, of point source *j*, and *δ*(**x** − **x**_*j*_) is the delta function. The term *ϕ*_*j*_(**x**)*δ*(**x** − **x**_*j*_) represents a point source of fluid flux from the vasculature to the surrounding interstitial domain.

Applying the substitution $\bar{p}=p-p_{\infty }$, the Green’s function, *G*(**x**, x*), for the adjoint problem for $\bar{p}$ is given by

$$-\kappa \nabla^{2}G=\delta (x-x^{*}),$$ (10a)

G→0as|x|→∞. (10b)

For a given source, we distribute the delta function over a sphere of finite radius, *r*_0_. We assume continuity of *G* and ∇*G* at the interface *r* = *r*_0_, which enables the following radially symmetric Green’s function to be derived:

$$G(r)=\{\begin{array}{ll} \frac{1}{8\pi \kappa r_{0}}\left[3-{\left(\frac{r}{r_{0}}\right)}^{2}\right], & \text{if}\;r\leq r_{0},\\ \frac{1}{4\pi \kappa r}, & \text{if}\;r>r_{0}. \end{array}$$ (11)

We distribute a total of *N*_*s*_ sources across the vasculature with a maximum spacing of *δ*, and assign a source radii, *r*_0*i*_, equal to the corresponding blood vessel radius (see Fig 1c). Using Green’s superposition principle for linear operators, the convolution of *G* provides the corresponding pressure solution for source *i* ∈ *N*_*s*_ and so

$$\begin{array}{c} p_{i}=p_{\infty }+\sum_{j=1}^{N_{S}}G_{ij}q_{j}^{s}\;\text{for}\;i\in N_{s}, \end{array}$$ (12)

where $q_{j}^{s}$ is the vector of source strengths. Here, *G*_*ij*_ is the Green’s function associated with (10) and defined by

$$G_{ij}=\{\begin{array}{ll} \frac{1}{8\pi \kappa_{i}r_{0i}}\left[3-{\left(\frac{r_{ij}}{r_{0i}}\right)}^{2}\right], & \text{if}\;r_{ij}\leq r_{0i},\\ \frac{1}{4\pi \kappa_{i}r_{ij}}, & \text{if}\;r_{ij}>r_{0i}, \end{array}$$ (13)

where *r*_*ij*_ is the distance between sources, defined as *r*_*ij*_ = |**x**_*i*_ − **x**_*j*_|, and *κ*_*i*_ is the interstitial hydraulic conductivity at the location of source *i*.

From (6), the Green’s function, *G*_*ij*_, can be used to calculate the volume-averaged IFV, given by

$$\begin{array}{c} u_{i}=-\kappa_{i}\sum_{j}\nabla G_{ij}q_{j}^{s}\;\text{for}\;i\in N_{s} \end{array}$$ (14)

where

$$\nabla G_{ij}=\frac{dG_{ij}}{dr}=\{\begin{array}{ll} -\frac{r}{4\pi \kappa_{i}r_{0i}^{3}}, & \text{if}\;r\leq r_{0i},\\ -\frac{1}{4\pi \kappa_{i}r_{ij}^{2}}, & \text{if}\;r>r_{0i}. \end{array}$$ (15)

Starling’s law, (8), can be rearranged into the form

$$\begin{array}{c} p_{v,i}=p_{b,i}-K_{i}J_{v,i}-\sigma_{i}\left(\Pi_{b,i}-\Pi_{v,i}\right)\;\text{for}\;i\in N_{s}, \end{array}$$ (16)

where *p*_*v*,*i*_ (calculated by (12)) and Π_*v*,*i*_ are the blood and oncotic plasma pressure at the vessel wall, *p*_*b*,*i*_ (calculated by (5)) and Π_*b*,*i*_ are the vascular blood pressure at source *i*, in the absence of diffusive interstitial fluid transfer, and oncotic fluid pressure, *J*_*v*,*i*_ is the rate of fluid flow per unit volume from blood vessel *i* to the interstitium *σ*_*i*_ is the oncotic reflection coefficient for vessel *i*. The intravascular resistance to fluid transport across blood vessel *i*, is defined by

$$\begin{array}{c} K_{i}=\frac{1}{L_{p,i}S_{i}}, \end{array}$$ (17)

where *S*_*i*_ and *L*_*p*,*i*_ is the surface area and vascular conductance of vessel *i*, respectively.

Integrating over the volume of vessel *i* and assuming flux at the interface is continuous, we define

$$\begin{array}{c} J_{v,i}=-2\pi \kappa_{i}r_{v,i}l_{i}\sum_{j=1}^{N_{s}}\nabla G_{ij}q_{j}^{s}, \end{array}$$ (18)

where *r*_*v*,*i*_ is the vessel radius and *l*_*i*_ is the length of vessel *i*.

Eqs (12), (16) and (18) are then combined to give a set of *N*_*s*_ equations to be solved for the source fluxes $q_{j}^{s}$,

$$\begin{array}{c} \sum_{j=1}^{N_{s}}(G_{ij}-\frac{\kappa_{i}}{L_{p,i}}\nabla G_{ij})q_{j}^{s}=p_{b,i}-p_{\infty }-\sigma_{i}\left(\Pi_{b,i}-\Pi_{v,i}\right). \end{array}$$ (19)

Prescribing parameter values (see Table 2), the resulting solutions for $q_{j}^{s}$ can be solved and used to update vascular pressures, *p*_*i*,*b*_, using Starling’s law, (8), in the absence of an oncotic pressure gradient (i.e. ΔΠ = 0), and (18). Here, for iteration *k* + 1, *p*_*i*,*b*_ is set equal to the IFP at wall of blood vessel *i*, *p*_*i*,*v*_, calculated on iteration *k*. This iterative system is repeated by updating $q_{j}^{s}$ values until tolerances are reached ($O(10^{-6})$ *μ*l/min), subsequently, tissue IFP and IFV fields can be computed using (12) and (14), respectively.

**Table 2:** Fluid transport parameters.

Parameter	Description	Value	Units	Reference
*p*_∞_	Far-field interstitial fluid pressure	0	mmHg	-
*κ*	Interstitial hydraulic conductivity	1.7 ⋅ 10^−7^	cm^2^ mmHg^−1^ s^−1^	[17]
*L*_*p*_	Vascular hydraulic conductance	2.8 ⋅ 10^−7^	cm mmHg^−1^ s^−1^	[11]
*σ*	Oncotic reflection coefficient	0.82	-	[72]
Π_*b*_	Oncotic pressure of blood	20	mmHg	[72]
Π_*v*_	Oncotic pressure at the vessel wall	15	mmHg	[11, 74]
*S*/*V*	Ratio of vascular surface area to tumour volume			
GL261		17.3	cm^−1^	Calculated.
LS147T		15.3	cm^−1^	Calculated.

Assigned baseline parameters for the interstitial component of the fluid transport model. Note, *S* was calculated using the architectural data of the vasculature and *V* based on computing the convex hull of the tumour.

In effect, our computational framework enables a detailed, quantitative assessment of blood and interstitial flow for tissues, both healthy and pathological, where their entire vascular networks are characterised, by encapsulating the flow of fluid between the vascular and interstitial domains. Similar to a Green’s function model for O2 transport [42], our model does not require the imposition of explicit boundary conditions on the outer surface of the tissue domain, with the only unknowns to the system being the strength of the set of fluid sources and sinks. As such, when compared to finite difference or element methods, our approach minimises boundary condition artefacts and saves on computational expense as the solutions to the entire mesh are not required. An outline of the interaction between the biomedical imaging, and vascular and interstitial flow compartments is given in Fig 1d.

The computational framework was coded using C++ [67] and run on a Apple Mac Pro, with 2 × 3.06 GHz 6-Core Intel Xeon processor and 64 GB of RAM. The system (19) was used solved using a biconjugate gradient method [68] and implemented using the Armadillo sparse linear algebra library [69].

### Implementation of computational model on whole tumour microvascular networks

**Fig 2:**
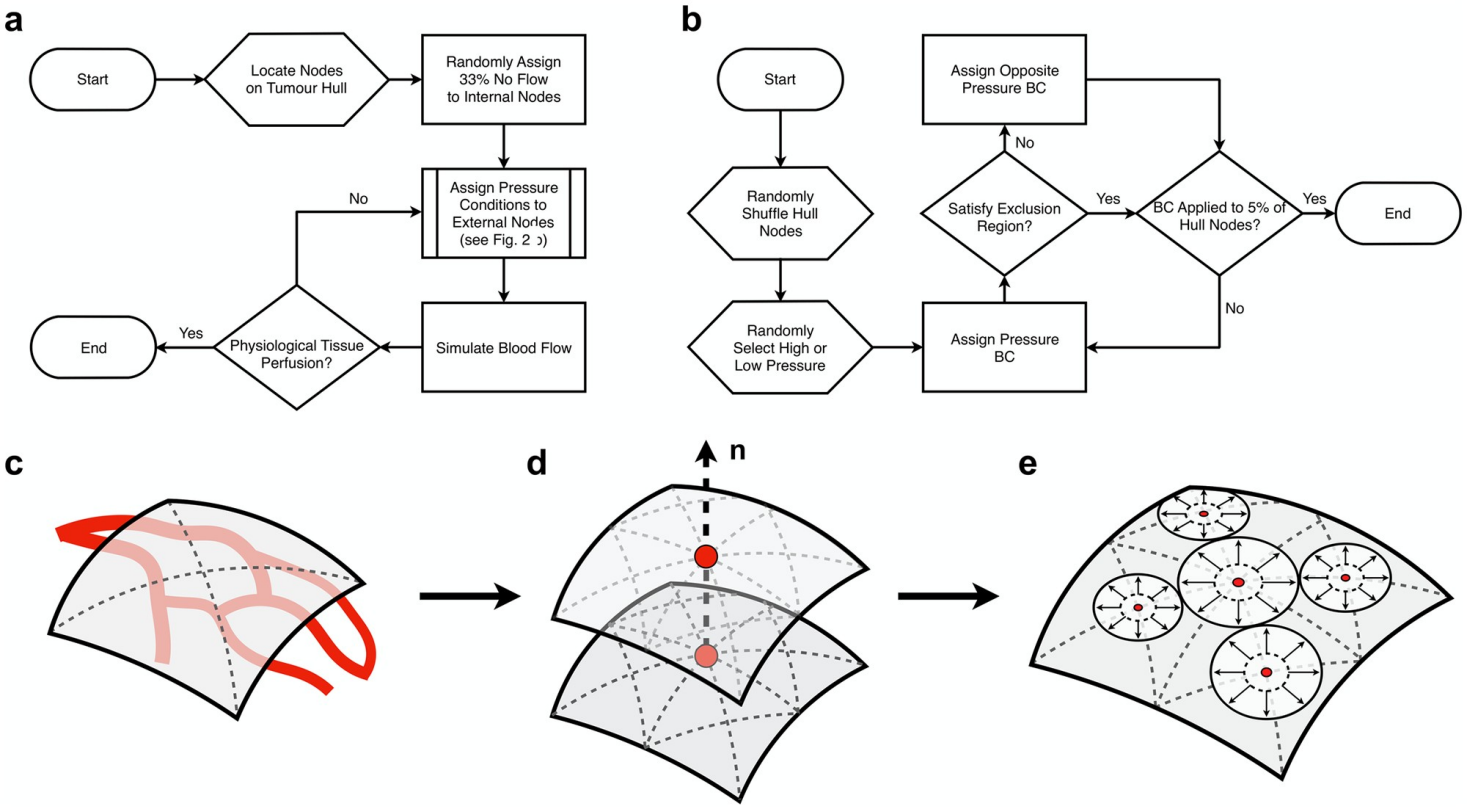
(a, b) The optimisation scheme used to assign boundary conditions to the tumour networks. (a) The process to simulate physiological tissue perfusion. (b) The flowchart for the subroutine “Assign Pressure Conditions” given in (a). (c) Perfusion through a tumour is calculated by generating a convex hull across the surface of the tumour to accurately extract tumour volume. (d) Discretising the hull into a finer mesh and calculating IFP at coupled points across, and normal, to the tumour hull. (e) A sphere packing algorithm is then applied to the points on the tumour surface with inflow averaged across the great circles of each sphere, enabling an approximation of perfusion.

It remains practically infeasible to measure vascular flows and pressures in individual microvessels *in vivo*, which necessitates a pragmatic approach to boundary condition assignment. Under the assumption that vessels along the tumour surface are connected to peritumoural vessels [37, 70], we developed an optimisation procedure which assigns vascular pressures to tumour surface vessels, based on a target pressure drop, with iterative adjustments to match *in vivo* measurements of mean perfusion from ASL-MRI (see Fig 2a and 2b). These *in vivo* data are acquired for the same tumours that were subsequently subjected to OPT analysis. Using this approach, we are able to ensure good agreement between *in silico* predictions and measured perfusion data [40].

In this study a vascular pressure of 30 or 20 mmHg for the GL261 and 45 or 15 mmHg for the LS147T tumour was randomly assigned to 5% of surface boundary nodes, in order to meet the required tissue perfusion. To ensure randomness, the peritumoural nodes were represented by a list. The elements in the list were rearranged randomly using a uniform random number generator where the system clock was used to seed the random number engine. The nodes located in the top 5% of the list were then randomly assigned a low or high pressure using an equivalent randomised approach.

During preliminary simulations it was found that if high/low pressures were prescribed in close local proximity to each other, unphysiological flows were predicted due to the steep local vascular pressure gradient. In order to prevent this, a subroutine was designed so that values at opposing ends of the pressure spectrum could not be located within a defined vicinity of each other. This “exclusion region”, centred on a given boundary node, was defined as an ellipsoid volume with diameters, along its three principal axes, equal to 5% of the tissue dimensions.

A proportion of boundary nodes are contained within the tumour volume as a consequence of either angiogenesis which form blind-end vessels, or artefacts of OPT. Previous studies approximated the fraction of blind-end vessels in sample MCa-IV carcinomas to be 33% [71]. However, blind-end information is not available for either GL261 or LS147T tumours. Therefore, consistent with previous computational studies [37], blind-ends were randomly applied to 33% of remaining boundary nodes (using the previous randomised approach), with the remaining 62% of boundary nodes left as unknown in the flow optimisation scheme of Fry et al. [41].

Application of our boundary assignment method requires us to accurately compute mean perfusion across the tumours for a comparison against equivalent experimental data gathered *in vivo* using ASL-MRI. This requires an accurate definition of the tumour surface and volume to give an accurate approximation of a tumour’s mass and fluid flow into the tumour volume. For example, an overestimation of the tumour mass, assuming a cuboid tissue volume surrounding the tumour, can drastically underestimate tumour perfusion since, in this case, the tumour shapes are approximately ellipsoidal and perfusion is inversely proportional to the tumour mass. Similarly, an overestimation of flow into the tissue would overestimate tissue perfusion. Next we describe: 1) defining the surface of the tumour; 2) computing the IFV vectors across the tumour surface; and 3) approximating the total tissue perfusion.

1) The hull of a tumour is calculated using the Matlab (MathWorks Inc., Natick, MA) ‘*boundary*’ function applied to all nodes defined during vascular segmentation (see Fig 2c). The Matlab ‘*fast loop mesh subdivision*’ triangulation algorithm is then applied to further discretise the define tumour surface. 2) To approximate tumour perfusion requires us to define a set of normal vectors to the tumour surface to compute pressure gradients. We identify the centre of the tumour and duplicate the hull, which is then expanded to form a 10 *μ*m gap between the two surfaces (see Fig 2d). IFP is then calculated across all nodal points on each surface, each paired by a vector normal to the opposing surface. A pressure gradient is then computed along each normal vector and the corresponding velocities are calculated. 3) A sphere packing algorithm is applied to the nodes on the original tumour hull, whereby no sphere overlaps neighbouring spheres (see Fig 2e). Any inflowing node (defined by the corresponding pressure gradient) is averaged across the great circle of its corresponding sphere and its contribution is summed together to calculate the interstitial component of tissue perfusion. Total tissue perfusion is calculated by summing over the peritumoural vascular inlets and interstitial perfusion values.

Finally, we prescribe baseline parameter values (see Table 2). We assume that the tumours were isolated in subcutaneous tissue in the absence of lymphatics, therefore the far-field pressure, *p*_∞_, was set to 0 mmHg. Due to a lack of experimental data, the vascular conductance and interstitial conductivity, *L*_*p*_ and *κ*, respectively, were given uniform values based on literature (2.8 × 10^−7^ cm mmHg^−1^ s^−1^ and 1.7 × 10^−7^ cm^2^ mmHg^−1^s^−1^, respectively [31, 75]). As the transport of blood plasma proteins is not modelled explicitly in our model, we assume a constant oncotic pressure gradient of 5 mmHg between the vasculature and interstitium, and set the oncotic reflection coefficient to a uniform value of 0.82 [72].

## Results

**Fig 3:**
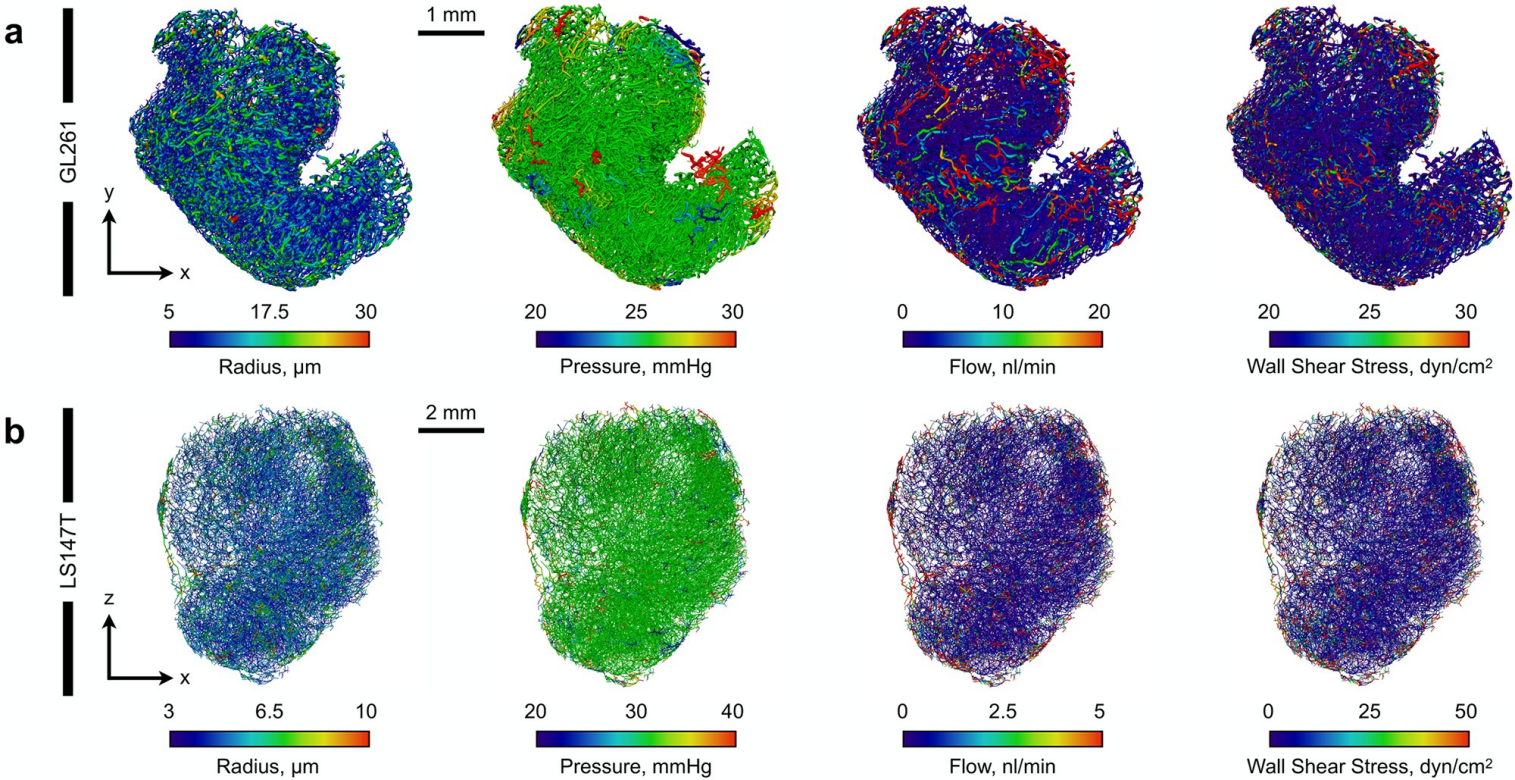
Simulated vascular blood flow in (a) GL261 and (b) LS147T tumours. Distributions are shown for vessel radii, blood pressure, flow and vessel wall shear stress, respectively.

**Fig 4:**
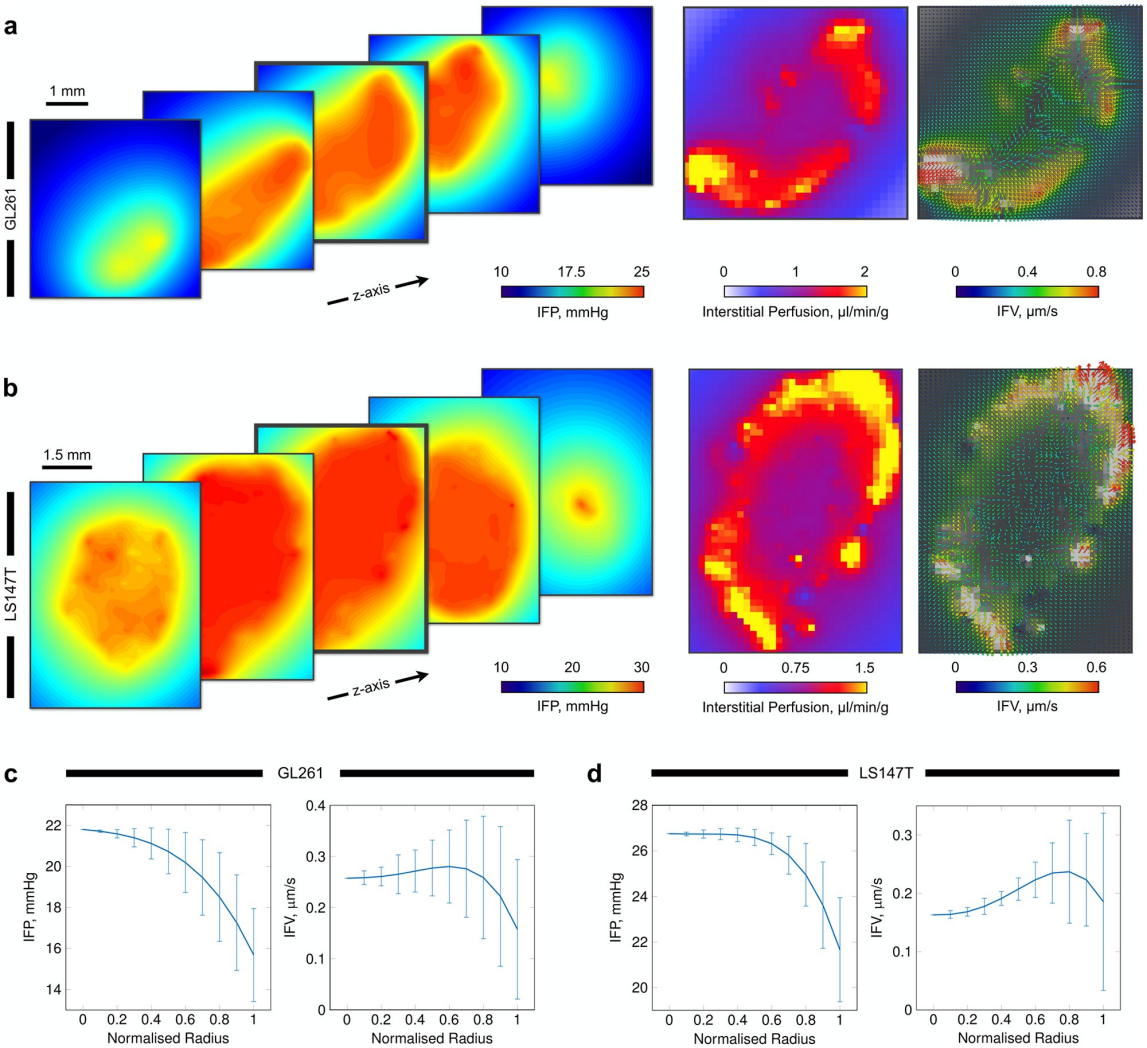
Simulated fluid transport through the interstitium in GL261 and LS147T tumours. (a, b) (Left) Predicted interstitial fluid pressure (IFP) fields for {X,Y}-planes through the tumours, emulating the traditionally high pressure in the tumour core but with predicted spatial heterogeneities. (Middle) Simulated interstitial perfusion maps discretised into ∼ 140 *μ*m^2^ pixels. Results replicate the traditionally elevated perfusion existing at the periphery of the tumours. (Right) Interstitial fluid velocity (IFV, overlaid onto greyscale image of interstitial perfusion) predictions depicting spatial interstitial flow heterogeneities across the entire tumours. Note, perfusion and interstitial fluid velocity maps are shown for the central slice in the interstitial fluid pressure graphics. (c, d) Fitted curves with error bars indicating standard deviation for (left) IFP and (right) IFV in (c) GL261 and (d) LS147T, plotted against normalised radius, corresponding to the simulations shown in (a, b).

In the following section we apply our computational framework to a GL261 orthotopic murine glioma and a LS147T human colorectal carcinoma xenograft to form baseline flow solutions (see Figs 3 and 4). Next, we explore sensitivity to source parameters, which include source distribution, source radii and bilateral communication between the vascular and interstitial compartments. We then perform sensitivity analysis to the interstitial parameters (for example, vascular hydraulic conductance and interstitial hydraulic conductivity) on IFP and IFV profiles in the LS147T tumour. This is followed by *in silico* experiments to establish the tumour parameters, such as vascular and interstitial permeability, which change tumour IFP and perfusion following vascular normalization therapy, and show how vascular architectural heterogeneities affect treatment response.

### Simulating interstitial fluid pressure in real-world cancerous tissue

**Fig 5:**
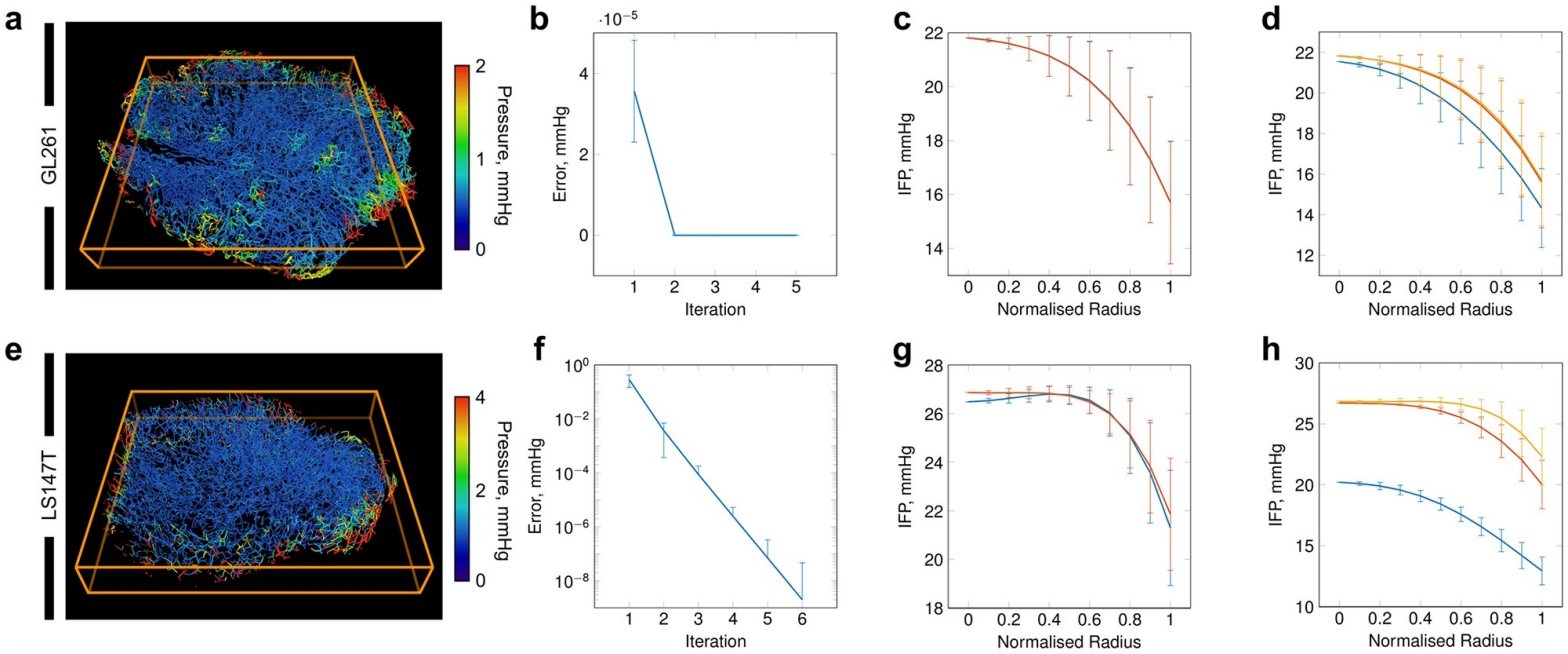
Sensitivity of the computational framework. (a, e) Cross-sectional slice from the core of the (top) GL261 and (bottom) LS147T tumours, showing standard deviation of intravascular pressures (mmHg) across all 12 simulations. (b, f) the mean segment pressure error between consecutive iterations for the (top) GL261 (top) and (bottom) LS147T networks. (c, g) IFP distribution from the centre of the tumour to its periphery for a maximum spacing of (blue) 10 and 25 *μ*m and (orange) 50 and 100 *μ*m for GL261 and LS147T, respectively. (d, h) IFP distributions for the source radii set to the minimum vessel radius multiplied by a factor of (blue) 10^−1^, (red) 10^0^ and (yellow) 10^1^. Note, error bars correspond to standard deviation.

Our vascular flow simulations are in good agreement with those in computational [37, 76] and experimental literature (see Table 3 and Fig 3). To test the variability induced by our stochastic boundary condition implementation, the optimisation procedure (detailed in Fig 2a and 2b) was repeated for a total of n = 12 for each tumour. Interstitial fluid flow was then simulated for each separate vascular flow solution, providing us with a baseline set of interstitial flow solutions. Blood flow across all simulations exhibited similar spatial distributions, with perfused vessels mainly restricted to the outer rim of the tumours [77]. The mean standard deviation of vascular blood pressures across all simulations was low with values of 0.82 and 1.53 mmHg (with maxima of 4.78 and 13.2 mmHg) for GL261 and LS147T, respectively (see Fig 5a and 5e). The elevated standard deviations in vascular pressure were located at the periphery of the tumours, which is to be expected as the high and low vascular pressures were stochastically assigned here. Furthermore, our mean blood velocity and vessel wall shear stresses agree with similar numerical modelling of vascular blood flow in the MDA-MB-231 breast cancer cell line [37].

**Table 3:** Simulated fluid transport statistics.

Parameter	GL261	LS147T	Literature	Units
Blood Pressure	25.0 ± 0.9	30.6 ± 2.0	10 − 80 [76]	mmHg
Blood Flow	5.1 ± 43.4	1.1 ± 4.7	0 − 180 [76]	nl min^−1^
Blood Velocity	0.2 ± 1.0	0.2 ± 0.6	1.62 ± 0.14 [37]	mm s^−1^
Vessel Wall Shear Stress	5.7 ± 23.2	11.8 ± 32.6	23.9 ± 0.7 [37]	dyn cm^−1^
Tissue Perfusion	83.7 ± 22.3	5.4 ± 0.3	110 ± 70^†^, 19 ± 8^‡^ [40]	ml min^−1^ 100 g^−1^
Interstitial Fluid Pressure (IFP)	20.4 ± 2.1	25.3 ± 1.6	13.5 ± 11.3 [74]^‡^	mmHg
Interstitial Fluid Velocity (IFV)	4.8 ± 0.5	0.19 ± 0.09	2.3 [25], 0 − 200‡ [26]	*μ*m s^−1^

Intra- and extravascular baseline fluid transport statistics for the GL261 and LS147T tumours (mean ± standard deviation) across all simulations and compared against literature values.

^†^: Data gathered from GL261 tumours.

^‡^: Data gathered from LS147T tumours.

Tissue perfusion (calculated as 83.7 ± 22.3 and 5.4 ± 0.3 ml/min/100g for the GL261 and LS147T tumours, respectively) was further validated by *in vivo* ASL-MRI measurements of 110 ± 70 and 19 ± 8 ml/min/100g for GL261 and LS147T, respectively. This implies that imposing physiologically realistic pressure boundary conditions generates physiologically realistic perfusion, and consequently accurate drug delivery solutions [40].

No literature IFP values were available for GL261 cell lines, however, our IFP was slightly elevated compared to that previously measured in LS147T *in vivo* (13.5 ± 11.3 mmHg [74]). Considering the range of IFP both here and *in vivo* [74] and the good accordance with *in vivo* perfusion, our results provided us with the confidence that our simulations can produce physiological IFP predictions (see Fig 4).

Examining the tumour radial IFP profiles, the LS147T network exhibited similar configurations as observed both experimentally in LS147T [17], in other cell lines [3, 24, 25], and in computational studies [31, 32, 36, 75], with elevated IFP at the tumour core (see Fig 4b and 4d). In addition, the LS147T network displayed a typical IFV profile radially, with an increasing IFV range towards the tumour surface due to the steeper pressure gradients at the periphery of the tumour. This indicated that bulk fluid filtration occurs at the high flowing vasculature located at the tumour extremity in this network (*ρ* = 0.41, *p* < 0.001, where *ρ* and *p* are the Pearson’s correlation coefficient and its corresponding *p*–value between tumour radius and vascular flow, respectively).

In comparison, the GL261 network also exhibited a traditional, yet steeper, IFP profile with a wider variance throughout the tumour (see Fig 4c). The IFV profile exhibited a typical profile increasing from the centre of the tumour to the periphery. However, its IFV peak was reached at ∼ 80% of the tumour radius, with a substantial decline in the latter 20% (see Fig 4c).

High IFP has been associated with low vascular density in A-07-GFP tumours [78]. Similarly here, we observed that GL261 and LS147T have distinct differences in their vascular architecture (see Fig 3 and Table 1) and that, in the case of LS147T, low variability in vascular density was associated with higher IFP (*ρ* = −0.584, *p* < 0.001—including *in silico* predictions from [40]). This may indicate that the inherent vascular architectural heterogeneity across tumour cell lines [20] directly impacts the IFP and IFV distributions, creating an unorthodox interstitial flow profile.

### Sensitivity of tumour interstitial fluid transport to model parameters

Here, we perform sensitivity analysis to understand the impact of parameter variance on predictions of tumour fluid dynamics. In doing so, not only do we understand the sensitivity to the model, but gain insight into the biological mechanisms at play.

For convenience we have split these parameters into two groups. The first we define as *source parameters*, which include the vascular blood pressure, *p*_*b*,*i*_ and the source radius, *r*_0,*i*_ for segment *i*, and the maximal spacing between each source along a given vessel, *δ* (see Fig 1c). The second group we call the *interstitial parameters*, which describe the biomechanical mechanisms which affect tissue transport. These include the oncotic reflection coefficient, *σ*, the hydraulic conductance of a vessel wall, *L*_*p*_, the interstitial hydraulic conductivity, *κ* and the far-field IFP, *p*_∞_. In the following, if sensitivity analysis to an interstitial parameter was not being performed, it was set to the corresponding value in Table 2.

#### Source parameters

Blood pressure distributions across a vascular network depend not only on the vascular pressure at the network boundaries and network geometry, but also transvascular fluid transport from the tumour vasculature and into the surrounding interstitium, and vice versa. To incorporate flow communication between these two domains, we couple the vascular and interstitial models using an iterative scheme in which vascular blood pressure distributions, *p*_*b*,*i*_ for *i* ∈ *N*_*s*_, were updated on each iteration by incorporating the loss of fluid within a vessel due to fluid flux across the vessel wall into the interstitium (see Fig 1d). We found that due to the small volume of fluid leaving the vessels (relative to the volume of fluid flowing through the vessels), vascular pressures did not vary significantly between iterations (see Fig 5b and 5f). In the case of the GL261 network, the algorithm had converged after two iterations (see Fig 5b). In comparison, the LS147T network had not converged after six iterations but the mean nodal pressure error was $O(10^{-8})$ mmHg (see Fig 5f). This may indicate that a tumours inherent vascular heterogeneity and corresponding interstitial parameters alter the scale of these flow errors.

Next we varied the maximum distance, Δ_*max*_, between sources distributed along the vasculature. Values of 10 and 25 *μ*m, and 50 and 100 *μ*m were chosen for the GL261 and LS147T networks, respectively, due to differences in mean branching vessel lengths observed in each tumour (see Table 1). The GL261 network exhibited minimal sensitivity in IFP between the two maximal source lengths (see Fig 5c). Comparatively, the LS147T experienced greater variability (of less than 1 mmHg) in mean IFP, between the two values of Δ, at the core and periphery of the tumour (see Fig 5g). We hypothesise that sensitivity to Δ is related to the vascular density of each tumour type. The GL261 network is an order of magnitude greater in vascular density compared to the LS147T tumour (see Table 1). An elevated vascular density results in an increased density of fluid sources, and so any reduction in sources may have a minimal effect to IFP due to flow being maintained by local neighbouring sources.

We finally sought to understand how the source radii, *r*_0,*i*_ for *i* ∈ *N*_*s*_, affect flow in the interstitial domain. We initially set the source radii to a constant value equal to the minimum vessel radius in a given tumour network. Three cases were then explored where *r*_0,*i*_ was multiplied by a factor of 10^−1^, 10^0^ or 10^1^. Our results show that decreasing the value of the source radii decreases mean IFP, with greater sensitivity exhibited again by the LS147T network (see Fig 5d and 5h), again indicating that vascular architectural heterogeneities can illicit different responses in IFP between tumours. This indicates that greater care is needed compared to the assignment of other source parameters, to ensure the physiological accuracy of the results. Here, we set the value of *r*_0,*i*_ equal to the radius of its corresponding vessel in order to provide a heterogeneous distribution of radii in which vessels with a larger radius are able to influence the IFP to a greater capacity than compared to relatively smaller vessels.

#### Interstitial parameters

**Fig 6:**
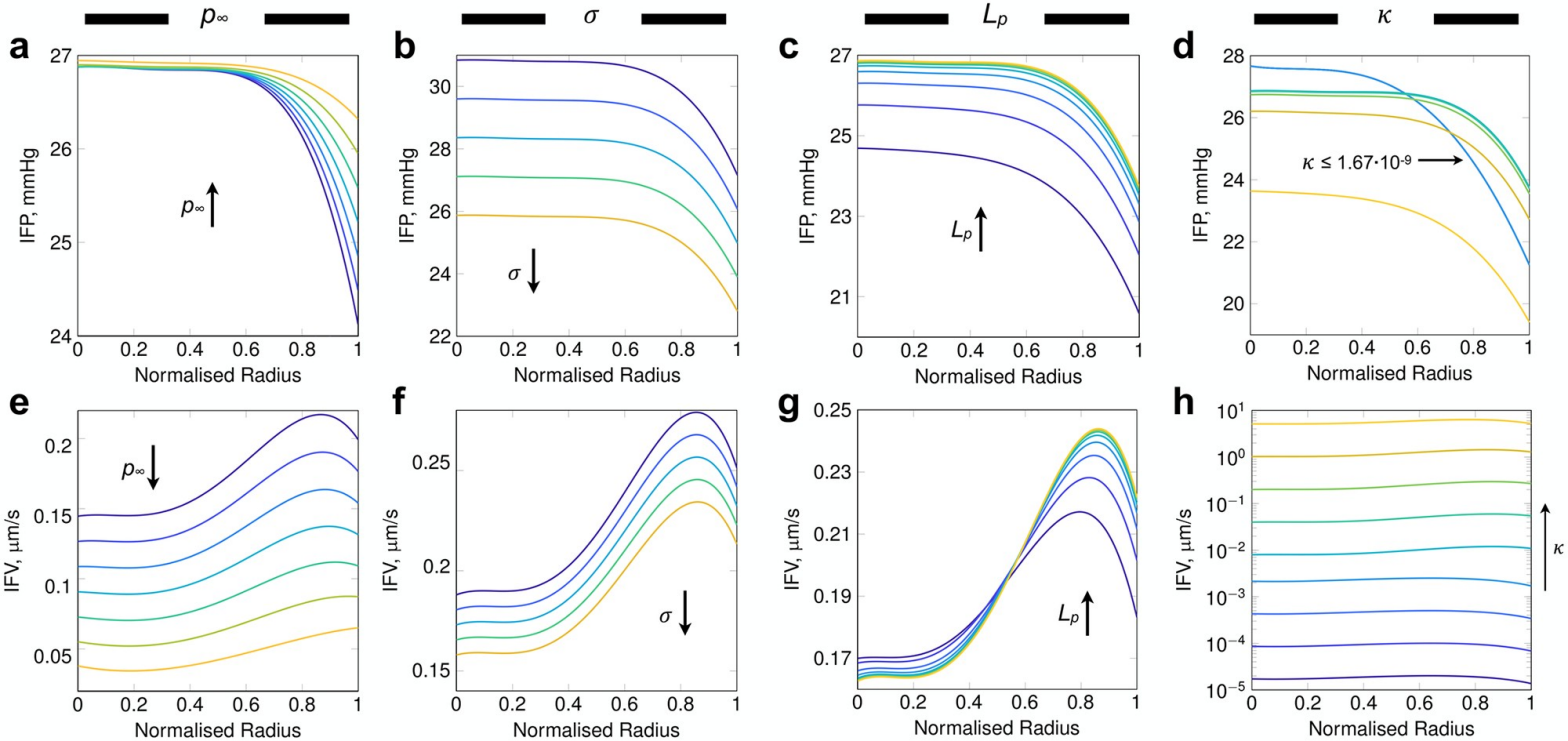
Data-fitted curves of IFP (top) and IFV (bottom) for modulation of interstitial model parameters: (a, e) *p*_∞_ (mmHg), (b, f) *σ*, (c, g) *L*_*p*_(cm / mmHg s) and (d, h) *κ* (cm^2^ / mmHg s). Arrows indicate increasing parameter values, with exception of (d) in which a range of *κ* (cm^2^ / mmHg s) is indicated. IFP profile gradients across LS_2_ sensitivity analysis of (a) far-field pressure, *p*_∞_, (b) oncotic reflection coefficient, *σ*, (c) vascular hydraulic conductivity, *L*_*p*_, and (d) interstitial hydraulic conductivity, *κ* (cm^2^ / mmHg s). Arrows indicate increasing values of the given parameter and colours in each column indicate the equivalent simulation.

Sensitivity to the interstitial parameters *p*_∞_, *σ*, *L*_*p*_, and *κ* were explored using the LS147T network. This network was chosen due to the scale of simulations to be performed and due to the greater sensitivity exhibited by modifying the source parameters. First of all, we investigated the deviation in the far-field interstitial pressure, *p*_∞_. Ideally, the far-field pressure is set to the mean IFP of the adjoining healthy tissue, however these data are frequently unavailable. We varied the far-field IFP from 3 to 18 mmHg in increments of 3 mmHg. Alteration of *p*_∞_ did not significantly modify tumour IFP, with the majority of variation occurring at the periphery of the tumour (see Fig 6a). As a consequence, mean IFV across the tumour decreased with increasing *p*_∞_ (see Fig 6e).

Next we investigated the oncotic reflection coefficient, *σ*. Physiologically, the coefficient *σ* varies between 0 and 1, indicating the likelihood that a molecule approaching a pore in the vessel lumen will be reflected back and thereby retained in the vascular compartment. As such, we ranged *σ* uniformly from 0 to 1 with increments of 0.25. Modification of *σ* resulted in a network-wide shift in tumour IFP magnitude, thereby preserving the IFP gradient (see Fig 6b). This is an intuitive result since we held the oncotic pressure gradient constant across the entire vasculature, therefore any increase in *σ* resulted in systematic reduction in both IFP and IFV (see Fig 6b and 6f).

Vascular hydraulic conductance, *L*_*p*_, defines the leakiness of the lumen to the transport of blood plasma. Our sensitivity analysis varied *L*_*p*_ uniformly across the tumour, from 1.33 × 10^−8^ to 3.41 × 10^−6^ cm/mmHg s, encompassing values provided in literature for normal and tumour tissue [11]. Similarly to *σ*, a reduction in *L*_*p*_ resulted in decreasing IFP across the tumour but with contrasting regional gradients (see Fig 6c). Our analysis showed that with decreasing *L*_*p*_, IFP tended towards the assigned far-field pressure due to decreasing fluid filtration from the vasculature, and so in its limit, the IFP would uniformly be equal to *p*_∞_. This is due to decreasing fluid filtration, indicating that the model produces a physiologically viable response. In comparison, a reduction in *L*_*p*_ induced minimal changes to IFV in the core of the tumour but resulted in decreases towards the periphery (see Fig 6g).

Interstitial hydraulic conductivity, *κ*, encapsulates the structure of the ECM, such as stiffness and density, and determines the permeability of the interstitium to fluid transport. Here, we ranged *κ* from 1.33 × 10^−11^ to 1.04 × 10^−6^ cm^2^/mmHg s uniformly through the tumour. In this case, we established that sensitivity to this parameter are non-trivial to elucidate. An inflexion point was observed for values 1.67 × 10^−9^ ≤ *κ* ≤ 8.33 × 10^−9^ cm^2^ / mmHg s in which the spatial IFP distribution switched from one configuration to another (see Fig 6d). For *κ* ≤ 1.67 × 10^−9^ cm^2^ / mmHg s, IFP predictions displayed near identical configurations (mean IFP of 25.8 ± 2.4 mmHg for all four cases) in which IFP displayed an increased gradient compared to greater values of *κ* along with decreasing IFV (see Fig 6h). In comparison, *κ* ≥ 8.33 × 10^−9^ cm^2^ / mmHg s displayed similar IFP profiles with mean IFP decreasing with increasing *κ* (26.2 ± 1.5 to 22.5 ± 1.6 mmHg).

In the next section we apply our computational model to predict changes in tumour fluid dynamics following vascular normalization therapy.

### Normalizing blood vessel lumen minimally reduces IFP

Vascular normalization is a therapeutic strategy used to restore tumour vasculature to a structural and functional state exhibited by healthy blood vessels [13, 15, 18, 49, 51, 79]. The impact of vascular normalization agents include a reduction in microvessel diameter, pruning of immature vessels, increased vascular maturity and pericyte coverage, and reduced vessel tortuosity [7, 51, 80]. As a result, normalization has been shown experimentally to lower tumour IFP [51, 52], enhance tumour vascular perfusion [52, 53] and improve drug delivery [48, 49, 81, 82]. However, the mechanistic links are missing which necessitates use of *in silico* modelling.

We next use our computational framework to investigate the effectiveness of vascular normalization in both GL261 and LS147T tumours where normalization therapy is administered intraperitoneally [48, 82]. We assume an ideal case in which vascular normalization has occurred in an axisymmetric manner whereby the effectiveness of the treatment increases from the tumour core to its periphery. To achieve this, the extent of stabilisation of vessel diameters and normalization of lumen permeability (through a combination of changes in *L*_*p*_ and *σ*), as a result of increase pericyte coverage, ranges linearly from typical tumour values in the core to physiological levels at the periphery. Here, vessel diameters were decreased by a factor of 1.99 [79], and set *L*_*p*_ and *σ* to 0.44 × 10^−7^ cm mmHg^−1^ s^−1^ and 0.91 [83], respectively.

**Fig 7:**
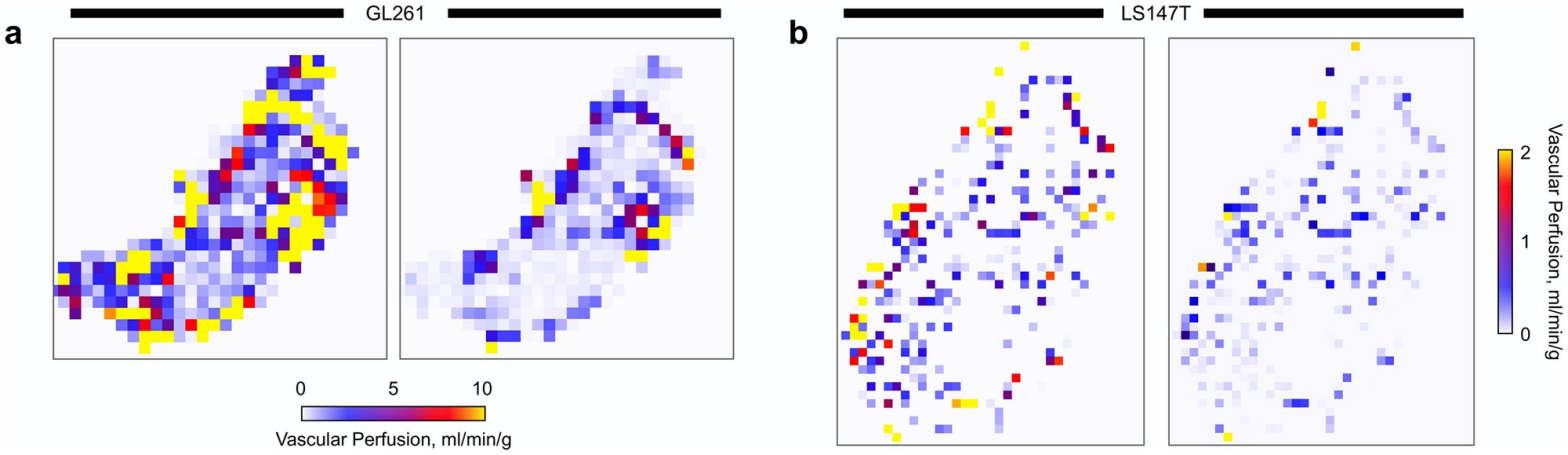
Predicting changes in vascular perfusion in response to vascular normalization therapy. Simulated vascular perfusion (left) pre- and (right) post-normalization in (a) GL261 and (b) LS147T tumours. Perfusion maps are discretised into isotropic pixels of width ∼ 140 *μ*m.

Similarly to experimental [51, 52] and computational [81, 84] studies, vascular normalization reduced vascular perfusion (see Fig 7) and mean IFP (see S1 Fig) across both the GL261 and LS147T tumours. However, our predictions showed that IFP remained elevated at the core and that the overall reduction was induced by an increase in the pressure gradient across the tumours. This resulted in a small increase in IFV at the core of both tumours (see S1 Fig), yet changes in interstitial perfusion were minimal.

Our therapeutic predictions of vascular normalization are inconsistent with the magnitude of observed effects, such as reduction in IFP, compared to experimentation [52, 53]. This suggests that normalizing the permeability of the vessel lumen and stabilising vessel diameters are not significant therapeutic affects following vascular normalization. We advocate that vascular normalization reduces tumour IFP and increases perfusion via other factors, such as changes in interstitial permeability or a reduction in vascular network tortuosity.

### Vascular normalization locally alters interstitial hydraulic conductivity

Vascular normalization fortifies blood vessels through an increase coverage of mural cells and basement membrane [85]. Considering this and our sensitivity analysis for *κ* (see Fig 6d and 6h), we hypothesise that normalization locally alters the interstitial hydraulic conductivity of a treated blood vessel. We therefore sought to normalize interstitial hydraulic conductivity, in parallel and in the same manner as with vessel diameters, *L*_*p*_ and *σ*, to a value of 8.53 × 10^−9^ cm^2^ mmHg^−1^ s^−1^ [86] at the tumour periphery.

**Fig 8:**
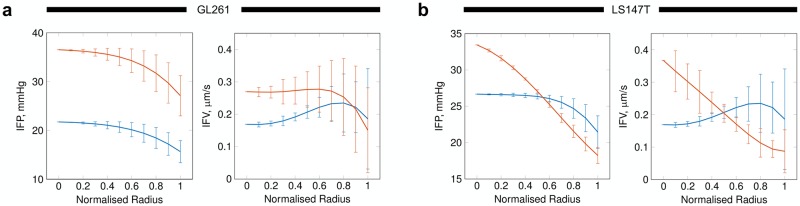
Simulated normlization of *L*_*p*_, *σ*, vessel diameters and *κ* in (a) GL261 and (b) LS147T tumours. Plots show (left) IFP and (right) IFV for (blue) pre- and (red) post-normalization. Error bars represent standard deviation.

**Fig 9:**
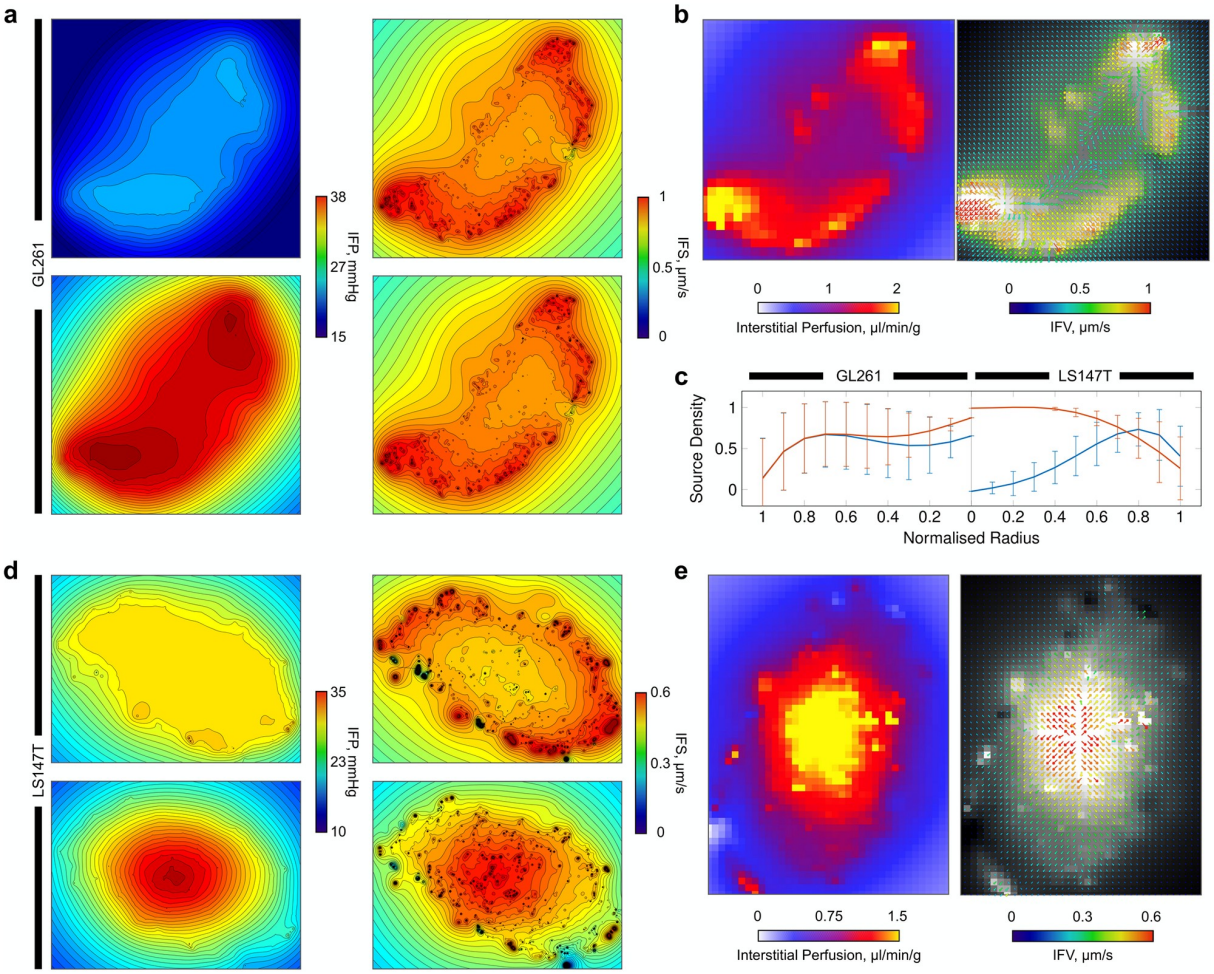
Normalization of vascular hydraulic conductance, oncotic reflection coefficient and interstitial hydraulic conductivity, and stabilisation of vessel diameters to physiological values in (a, b) GL261 and (d, e) LS147T tumours. (a, d) Planar contour plots of IFP and interstitial fluid speed (IFS) for the baseline (top) and normalised (bottom) predictions. (b, e) Predictions of normalised interstitial fluid spatial maps (left) and IFV (right—with greyscale perfusion underlaid) where the isotropic pixels are ∼ 140 *μ*m wide. For comparison, equivalent {X,Y}-planes for baseline simulations can be viewed in Fig 4a and 4b, respectively. (c) A comparison of change in the source density, the ratio between sources and sinks (= [*n* − *m*]/[*n* + *m*] where *n* and *m* are the sum of sources and sinks, respectively, in the tumour region), across GL261 and LS147T (blue) pre- and (orange) post-normalization.

Normalizing interstitial hydraulic conductivity had substantial and contrasting consequences to fluid transport in the interstitium of both GL261 and LS147T tumours. In the case of GL261, IFP was elevated compared to baseline simulations throughout the tumour (see Figs 8a and 9a), with a mean increase of 70%. This, in conjunction with a small increase in the IFP gradient, led to elevated IFV in 80% of the tumour (see Figs 8a and 9a) and consistent patterns in tumour perfusion compared to baseline (see Figs 4a and 9b).

Treating LS147T significantly modified the IFP profile (see Fig 9d), elevating IFP by ∼ 9 mmHg at its core and developing a steeper gradient towards the periphery, in addition to a narrowing of IFP standard deviation (see Fig 8b). As a result, IFV increased throughout the tumour core (see Fig 9d), with an increase of 50% at its centre, and a predicted decreased compared to baseline in the outer 50% of the tumour (see Fig 8b).

To understand the response to treatment we analysed the source density, the ratio between sources and sinks of fluid flux, across each tumour type pre- and post-normalization. Fig 9c shows that GL261 is dominated by sources of flux pre-treatment, with the number of sources elevating at its core post-treatment. In comparison, a parity is observed between sources and sinks in the core of LS147T pre-treatment. Following normalization, the balance between sources and sinks drastically altered with no sinks existing between 0 to 0.3 of the LS147T radius, this is followed by a decline in the ratio to levels lower that baseline at the periphery, indicating an increase of sinks here.

Our results suggest that observed fluid dynamic changes following vascular normalization in literature [51, 52, 52, 53] may be as a result of local modification of the interstitial hydraulic conductivity (see S2 Fig). Further, that changes in perfusion may be a result of an steepening of the IFP gradient across a tumour and not necessarily a uniform reduction in IFP across the tumour. This suggests the importance of incorporating inherent spatial heterogeneities in vascular networks observed across tumour cell-lines [20] (see Table 1 and S3 Fig) into *in silico* studies of vascular normalization.

## Discussion

Elevated interstitial fluid pressure is frequently associated with solid tumours, where a conventional profile exhibits a uniformly high IFP in the core of a tumour decreases rapidly towards the levels of physiological tissue at the periphery [3, 14, 65]. This atypical characteristic forms a barrier to transvascular fluid and drug delivery, thereby diminishing therapeutic efficacy of anti-cancer treatments.

The passage of fluid through the interstitium is influenced by both hydrostatic and oncotic pressures in blood vessels and therefore by the heterogeneous architecture of tumour microvessels. However, the procurement of detailed fluid flow data *in vivo* across whole tumour networks is currently infeasible using conventional imaging and experimental techniques. However, recent advances in *ex vivo* high-resolution optical imaging techniques [39] allow whole three-dimensional tissue architectures to be extracted and reconstructed to act as inputs for detailed *in silico* modelling of fluid transport.

No study has explicitly modelled vascular and interstitial fluid flow using discrete, three-dimensional structural data from images of whole-tumour, real-world vasculature [6]. However, in a recent study, we presented our novel REANIMATE platform which extracts three-dimensional, whole tumour vasculature *ex vivo* from optically cleared tissue, which is then used to parameterise an *in silico* model of fluid transport guided by *in vivo* imaging data [40]. This has enabled us to perform quantitative, realistic predictions of fluid and drug delivery to tumours which has led to novel insights into a tumour’s inherent physical resistance to anti-cancer therapies [40].

We present a generalised framework to model vascular and interstitial fluid dynamics based on whole, explicit tumour vasculature, in a computationally tractable way. We detail its derivation and application to whole tumour vascular datasets and show how our model allows highly-detailed predictions of fluid flow within the tumour microenvironment by incorporating explicit tumour vasculature and spatially heterogeneous parameters, such as vascular and interstitial permeability. Our model allows flow heterogeneities to be quantified in a computationally efficient manner, when compared to finite-difference and element methods [42], and consequently can be applied to any vascular tissue.

We initially apply our framework to an orthotopic murine glioma and a human colorectal carcinoma xenograft from the GL261 and LS147T cell-lines, respectively, to present realistic, baseline simulations of the tumour microenvironment. We then perform sensitivity analysis to the underlying model parameters. The first are the source parameters, which are specific to our model, and include point source distribution and size. Secondly, the interstitial parameters, such as vascular conductance and interstitial conductivity, which are frequently represented in literature due to the prominent use of Starling’s law. In the second case, we perform analysis of how variation of these parameters modifies the IFP and IFV in an LS147T dataset. We finally mimic vascular normalization therapy, via varying model parameters, to generate hypotheses relating to fluid dynamic response observed in literature.

Our computational framework is based upon a Poiseuille model for vascular blood flow [41] which is coupled to a steady-state Green’s function solution to interstitial fluid flow. Here, tumour vasculature segmented *ex vivo* is represented by a discrete set of sources of fluid flux for bi-directional transport between the interstitium. Previous models either assume a homogeneous vascular network [3, 11, 14, 65], incorporate a computer-generated synthetic tumour network [28–34], or incorporate boundary conditions and spatial variations in tissue permeability to artificially represent vascular heterogeneity [35, 36]. However, vascular averaging methods do not fully encapsulate the intrinsic, local interactions between neighbouring blood vessels which contribute to global interstitial flow and synthetic networks are difficult to validate against real tumour architecture. Here we use vascular architecture from real, whole tumour networks, and through use of Green’s function methods, our model significantly reduces the computational size of the computational problem, allowing vessel-vessel interactions to be modelled at the micron-scale. Thus, we provide the means to perform *in silico* studies to hypothesis test the impact of vascular heterogeneity on the tumour microenvironment with relative ease.

Our simulations were performed on *ex vivo* structural imaging data from a GL261 and a LS147T tumour. As no *in vivo* flow or pressure data were available for the numerous boundary vessels, we developed a procedure whereby simulated data is optimised based on *in vivo* tissue perfusion data gathered using ASL-MRI [40]. This approach has produced solutions which are highly consistent with experimental measurements in the same tumours [40]. In this study, baseline vascular flow solutions across all tumour simulations are in good agreement with the perfusion data, alongside mean flows [76], velocities and vessel wall shear stresses [37], and fluid pressure in the interstitium [3, 17, 24, 25]. This provided validation that our model produces physiologically realistic results, providing a platform to investigate the tumour microenvironment.

We performed sensitivity analysis to the source parameters, such as updating the vascular flow solution, source distribution and source radii, to understand their influence on the flow communication between the vascular and interstitial domains. Our results exhibited a minimal sensitivity to IFP distributions by varying these parameters, with the exception of the assignment of source radii. Here, we hypothesise that greater care is required for spatially sparse tumours with a low vascular density, in order to ensure physiologically accurate simulations. Investigating the sensitivity to the interstitial parameters in the LS147T tumour, we found that raising the far-field interstitial pressure did not significantly alter the IFP distribution across the tumour, with increased IFP occurring at the tumour periphery, compared to baseline. Uniform variation in the oncotic pressure contribution, by modifying the oncotic reflection coefficient, only affected the magnitude of the interstitial pressure, with minor changes to the IFP and IFV profiles, similar to those reported in previous studies [2]. Increasing *L*_*p*_ and *κ*, raised the gradient of the interstitial pressure profile, thereby increasing fluid transport through the interstitium. However, our IFP distributions did not reach 0 mmHg at the periphery of the tumours as in previous studies [3, 14, 84]. This is due to not applying a fixed pressure at the tumour boundary in our model, which results in a smoother transition of IFP to the surrounding tissue, similar to previous computational modelling of tumour vascular heterogeneity [35, 36].

We next predicted the flow response following vascular normalization therapy mimicked by varying model parameters. Normalization of tumour vasculature was achieved by modifying vascular hydraulic conductances, oncotic reflection coefficients and stabilising blood vessel diameters in a linear radially varying fashion in both tumour networks. Our results indicate that whilst normalisation of lumen permeability and vessel diameters exhibited similar trends compared to experimentation such as a reduction in vascular perfusion post-treatment [52, 53], the magnitude in IFP reduction was minimal. Similarly, therapy which soley reduces vessel leakiness has been shown not to be effective *in silico* using synthetic tumour vasculatures [34].

As vascular normalization has been shown to stabilise the tumour vasculature by increasing mural cell and basement membrane coverage [85], we hypothesise that these structural changes alter the interstitial hydraulic conductivity. Subsequent simulations observed significant and unique changes in IFP, IFV and perfusion across each tumour cell-line. In GL261, we predicted an increase in IFP across the entire tumour, whereas in LS1247T, IFP exhibited a steep pressure gradient and elevated IFP in its core. Consequently, IFV was elevated in both tumour cores. We noted that this significantly altered the interstitial perfusion map in LS147T, with elevated perfusion at the tumour core, which dissipated towards its periphery.

We have shown that incorporating realistic tumour vasculature is key to accurately predicting the spatial flow heterogeneities induced by blood vessels. Our results suggest that reducing tumour IFP is not necessary to increase tumour interstitial perfusion and that developing a IFP gradient across the tumour is the key response. Further, we hypothesise that vascular normalization alters interstitial hydraulic conductivity within tumours. However, as our tumour vascular networks are static, we cannot discern the relative impact caused by a reduction in vascular tortuosity observed experimentally following therapy [51, 80]. Notwithstanding, we show that normalizing the tumour interstitial environment may provide an effective means to increase tumour perfusion.

Our results and proposed computational framework offer significant scope for future expansion. For example, recent *in vivo* methods provide a step forward in approximating conditions by measuring interstitial fluid velocity at the macro-scale [26]. These data could lead to greater accuracy when assigning boundary conditions specific to a tumour. Further, parameter values such as the vascular conductance and interstitial hydraulic conductivity were assigned using previous literature values since these tissue-specific measurements can be challenging to procure through experimentation. For example, interstitial conductivity values across healthy tissue have been reported to span four orders of magnitude [17]. New methods need to be developed to accurately quantify these values.

There are also opportunities to expand the computational model to incorporate more complex biological phenomena. For example, incorporating tumour compression of vessels due to increasing shear stresses within the tumour [29, 87], volumetric tissue growth and tumour angiogenesis which would allow us to develop vascular normalization treatment strategies which determine when to administer therapy during the normalization window [12].

We expect to find a wide utility for REANIMATE in a range of disease areas, particularly given the current interest in optical clearing methods and their widespread use in biomedical research. Our *in silico* framework is novel and timely and will find extensive use for hypothesis testing, to enable tumour biology and drug delivery to be better understood, which in turn may enable the next generation of cancer therapies.

## Supporting information

S1 Fig Simulated normlization of *L*_*p*_, *σ* and vessel diameters in (a) GL261 and (b) LS147T tumours. Plots show (left) IFP and (right) IFV for (blue) pre- and (red) post-normalization. Error bars represent standard deviation.
(TIF)

S2 Fig Simulating normalization of interstitial hydraulic conductivity, *κ*, in (a) GL261 and (b) LS147T tumours. Plots show (left) IFP and (right) IFV for normalization of (blue) *L*_*p*_, *σ*, vessel diameters and *κ*, and (red) *κ*. Error bars represent standard deviation.
(TIF)

S3 Fig Vascular density across (a) GL261 and (b) LS147T tumours. Error bars represent standard deviation.
(TIF)

## References

[1] Minchinton AI, Tannock IF. Drug penetration in solid tumours. Nature Reviews Cancer. 2006;6(8):583–592. 10.1038/nrc1893 16862189

[2] Jain RK. Determinants of Tumor Blood Flow: A Review. Cancer Research. 1988;48(10):2641–2658. 3282647

[3] Baxter LT, Jain RK. Transport of fluid and macromolecules in tumors. I. Role of interstitial pressure and convection. Microvascular Research. 1989;37(1):77–104. 10.1016/0026-2862(89)90074-5 2646512

[4] Jain RK. Barriers to drug delivery in solid tumors. Scientific American. 1994;271(1):58–65. 10.1038/scientificamerican0794-58 8066425

[5] Jang SH, Wientjes MG, Lu D, Au JLS. Drug delivery and transport to solid tumors. Pharmaceutical research. 2003;20(9):1337–50. 10.1023/A:1025785505977 14567626

[6] Rieger H, Welter M. Integrative models of vascular remodeling during tumor growth. Wiley Interdisciplinary Reviews: Systems Biology and Medicine. 2015;7(3):113–129. 10.1002/wsbm.1295 25808551 PMC4406149

[7] Dewhirst MW, Secomb TW. Transport of drugs from blood vessels to tumour tissue. Nature Reviews Cancer. 2017;17(12):738–750. 10.1038/nrc.2017.93 29123246 PMC6371795

[8] Koumoutsakos P, Pivkin I, Milde F. The Fluid Mechanics of Cancer and Its Therapy. Annual Review of Fluid Mechanics. 2013;45(1):325–355. 10.1146/annurev-fluid-120710-101102

[9] Goel S, Duda DG, Xu L, Munn LL, Boucher Y, Fukumura D, et al Normalization of the Vasculature for Treatment of Cancer and Other Diseases. Physiological Reviews. 2011;91(3):1071–1121. 10.1152/physrev.00038.2010 21742796 PMC3258432

[10] Narang AS, Varia S. Role of tumor vascular architecture in drug delivery. Advanced Drug Delivery Reviews. 2011;63(8):640–658. 10.1016/j.addr.2011.04.002 21514334

[11] Jain RK. Transport of molecules across tumor vasculature. Cancer and Metastasis Reviews. 1987;6(4). 10.1007/BF00047468 3327633

[12] Jain RK. Normalizing Tumor Microenvironment to Treat Cancer: Bench to Bedside to Biomarkers. Journal of Clinical Oncology. 2013;31(17). 10.1200/JCO.2012.46.3653 PMC3731977 23669226

[13] Mitchell MJ, Jain RK, Langer R. Engineering and physical sciences in oncology: Challenges and opportunities. Nature Reviews Cancer. 2017;17(11):659–675. 10.1038/nrc.2017.83 29026204 PMC5683724

[14] Jain RK, Baxter LT. Mechanisms of heterogeneous distribution of monoclonal antibodies and other macromolecules in tumors: significance of elevated interstitial pressure. Cancer Research. 1988;48:7022–32. 3191477

[15] Jain RK. Antiangiogenesis Strategies Revisited: From Starving Tumors to Alleviating Hypoxia. Cancer Cell. 2014;26(5):605–622. 10.1016/j.ccell.2014.10.006 25517747 PMC4269830

[16] Heldin CH, Rubin K, Pietras K, Östman A. High interstitial fluid pressure—An obstacle in cancer therapy. Nature Reviews Cancer. 2004;4(10):806–813. 10.1038/nrc1456 15510161

[17] Boucher Y, Brekken C, Netti PA, Baxter LT, Jain RK. Intratumoral infusion of fluid: estimation of hydraulic conductivity and implications for the delivery of therapeutic agents. British Journal of Cancer. 1998;78(11):1442 10.1038/bjc.1998.705 9836476 PMC2063228

[18] Fukumura D, Kloepper J, Amoozgar Z, Duda DG, Jain RK. Enhancing cancer immunotherapy using antiangiogenics: opportunities and challenges. Nature reviews Clinical oncology. 2018;15(5):325–340. 10.1038/nrclinonc.2018.29 29508855 PMC5921900

[19] Pietras K, Rubin K, Sjöblom T, Buchdunger E, Sjöquist M, Heldin CH, et al Inhibition of PDGF receptor signaling in tumor stroma enhances antitumor effect of chemotherapy. Cancer Research. 2002;62(19):5476–5484. 12359756

[20] Folarin AA, Konerding MA, Timonen J, Nagl S, Pedley RB. Three-dimensional analysis of tumour vascular corrosion casts using stereoimaging and micro-computed tomography. Microvascular research. 2010;80(1):89–98. 10.1016/j.mvr.2010.03.007 20303995 PMC4398341

[21] Boucher Y, Baxter LT, Jain RK. Interstitial Pressure Gradients in Tissue-isolated and Subcutaneous Tumors: Implications for Therapy. Cancer Research. 1990;50(15):4478–4484. 2369726

[22] Gutmann R, Leunig M, Feyh J, Goetz AE, Messmer K, Kastenbauer E, et al Interstitial hypertension in head and neck tumors in patients: correlation with tumor size. Cancer research. 1992;52(7):1993–5. 1551128

[23] Nathanson SD, Nelson L. Interstitial fluid pressure in breast cancer, benign breast conditions, and breast parenchyma. Annals of Surgical Oncology. 1994;1(4):333–338. 10.1007/BF03187139 7850532

[24] Liu LJ, Schlesinger M. MRI contrast agent concentration and tumor interstitial fluid pressure. Journal of Theoretical Biology. 2016;406:52–60. 10.1016/j.jtbi.2016.06.027 27343032

[25] Jian Liu L, Brown SL, Ewing JR, Ala BD, Schneider KM, Schlesinger M, et al Estimation of Tumor Interstitial Fluid Pressure (TIFP) Noninvasively. PLoS ONE. 2016;11(7):e0140892 10.1371/journal.pone.0140892 27467886 PMC4965107

[26] Walker-Samuel S, Roberts TA, Ramasawmy R, Burrell JS, Johnson SP, Siow BM, et al Investigating low-velocity fluid flow in tumors with convection-MRI. Cancer Research. 2018;78(7):1859–1872. 10.1158/0008-5472.CAN-17-1546 29317434 PMC6298581

[27] Netti PA, Baxter LT, Boucher Y, Skalak R, Jain RK. Time-dependent Behavior of Interstitial Fluid Pressure in Solid Tumors: Implications for Drug Delivery. Cancer Res. 1995;55(22):5451–5458. 7585615

[28] Welter M, Rieger H. Physical determinants of vascular network remodeling during tumor growth. Eur Phys J E. 2010;33:149–163. 10.1140/epje/i2010-10611-6 20607341

[29] Vavourakis V, Wijeratne PA, Shipley R, Loizidou M, Stylianopoulos T, Hawkes DJ. A Validated Multiscale In-Silico Model for Mechano-sensitive Tumour Angiogenesis and Growth. PLoS Computational Biology. 2017;13(1):e1005259 10.1371/journal.pcbi.1005259 28125582 PMC5268362

[30] Stylianopoulos T, Soteriou K, Fukumura D, Jain RK. Cationic Nanoparticles Have Superior Transvascular Flux into Solid Tumors: Insights from a Mathematical Model. Annals of Biomedical Engineering. 2013;41(1):68–77. 10.1007/s10439-012-0630-4 22855118 PMC3886728

[31] Soltani M, Chen P. Numerical Modeling of Interstitial Fluid Flow Coupled with Blood Flow through a Remodeled Solid Tumor Microvascular Network. PLoS ONE. 2013;8(6):e67025 10.1371/journal.pone.0067025 23840579 PMC3694139

[32] Sefidgar M, Soltani M, Raahemifar K, Sadeghi M, Bazmara H, Bazargan M, et al Numerical modeling of drug delivery in a dynamic solid tumor microvasculature. Microvascular Research. 2015;99:43–56. 10.1016/j.mvr.2015.02.007 25724978

[33] Mohammadi M, Chen P. Effect of microvascular distribution and its density on interstitial fluid pressure in solid tumors: A computational model. Microvascular Research. 2015;101:26–32. 10.1016/j.mvr.2015.06.001 26093178

[34] Welter M, Rieger H. Interstitial Fluid Flow and Drug Delivery in Vascularized Tumors: A Computational Model. PLoS ONE. 2013;8(8). 10.1371/journal.pone.0070395 PMC3734291 23940570

[35] Zhao J, Salmon H, Sarntinoranont M. Effect of heterogeneous vasculature on interstitial transport within a solid tumor. Microvascular Research. 2007;73(3):224–236. 10.1016/j.mvr.2006.12.003 17307203

[36] Pishko GL, Astary GW, Mareci TH, Sarntinoranont M. Sensitivity Analysis of an Image-Based Solid Tumor Computational Model with Heterogeneous Vasculature and Porosity. Annals of Biomedical Engineering. 2011;39(9):2360–2373. 10.1007/s10439-011-0349-7 21751070 PMC3373181

[37] Stamatelos Sk, Kim E, Pathak AP, Popel SA. A bioimage informatics based reconstruction of breast tumor microvasculature with computational blood flow predictions. Microvascular Research. 2014;91:8–21. 10.1016/j.mvr.2013.12.003. 24342178 PMC3977934

[38] Stamatelos SK, Bhargava A, Kim E, Popel AS, Pathak AP. Tumor Ensemble-Based Modeling and Visualization of Emergent Angiogenic Heterogeneity in Breast Cancer. Scientific Reports. 2019;9(1):5276 10.1038/s41598-019-40888-w 30918274 PMC6437174

[39] Walls JR, Sled JG, Sharpe J, Henkelman RM. Resolution improvement in emission optical projection tomography. Physics in Medicine and Biology. 2007;52(10):2775–2790. 10.1088/0031-9155/52/10/010 17473351

[40] D’Esposito A, Sweeney PW, Ali M, Saleh M, Ramasawmy R, Roberts TA, et al Computational fluid dynamics with imaging of cleared tissue and of in vivo perfusion predicts drug uptake and treatment responses in tumours. Nature Biomedical Engineering. 2018;2(10):773–787. 10.1038/s41551-018-0306-y 31015649

[41] Fry BC, Lee J, Smith NP, Secomb TW. Estimation of Blood Flow Rates in Large Microvascular Networks. Microcirculation. 2012;19:530–538. 10.1111/j.1549-8719.2012.00184.x 22506980 PMC3407827

[42] Secomb TW, Hsu R, Park EYH, Dewhirst MW. Green’s Function Methods for Analysis of Oxygen Delivery to Tissue by Microvascular Networks. Annals of biomedical engineering. 2004;32(11):1519–1529. 10.1114/B:ABME.0000049036.08817.44 15636112

[43] Jain RK. Normalization of Tumor Vasculature: An Emerging Concept in Antiangiogenic Therapy. Science. 2005;307 (5706). 10.1126/science.1104819 15637262

[44] Huang Y, Goel S, Duda DG, Fukumura D, Jain RK. Vascular normalization as an emerging strategy to enhance cancer immunotherapy. Cancer Research. 2013;73(10):2943–2948. 10.1158/0008-5472.CAN-12-4354 23440426 PMC3655127

[45] Jain RK. Normalizing tumor vasculature with anti-angiogenic therapy: A new paradigm for combination therapy. Nature Medicine. 2001;7(9):987–989. 10.1038/nm0901-987 11533692

[46] Goel S, Wong AHK, Jain RK. Vascular normalization as a therapeutic strategy for malignant and nonmalignant disease. Cold Spring Harbor perspectives in medicine. 2012;2(3):a006486 10.1101/cshperspect.a006486 22393532 PMC3282493

[47] Jain RK, Duda DG, Willett CG, Sahani DV, Zhu AX, Loeffler JS, et al Biomarkers of response and resistance to antiangiogenic therapy. Nature Reviews Clinical Oncology. 2009;6(6):327–38. 10.1038/nrclinonc.2009.63 19483739 PMC3057433

[48] Park JS, Kim IK, Han S, Park I, Kim C, Bae J, et al Normalization of Tumor Vessels by Tie2 Activation and Ang2 Inhibition Enhances Drug Delivery and Produces a Favorable Tumor Microenvironment. Cancer Cell. 2016;30(6):953–967. 10.1016/j.ccell.2016.10.018 27960088

[49] Winkler F, Kozin SV, Tong RT, Chae SS, Booth MF, Garkavtsev I, et al Kinetics of vascular normalization by VEGFR2 blockade governs brain tumor response to radiation: role of oxygenation, angiopoietin-1, and matrix metalloproteinases. Cancer cell. 2004;6(6):553–63. 10.1016/j.ccr.2004.10.011 15607960

[50] Li W, Quan YY, Li Y, Lu L, Cui M. Monitoring of tumor vascular normalization: The key points from basic research to clinical application. Cancer Management and Research. 2018;10:4163–4172. 10.2147/CMAR.S174712 30323672 PMC6175544

[51] Tong RT, Boucher Y, Kozin SV, Winkler F, Hicklin DJ, Jain RK. Vascular normalization by vascular endothelial growth factor receptor 2 blockade induces a pressure gradient across the vasculature and improves drug penetration in tumors. Cancer research. 2004;64(11):3731–6. 10.1158/0008-5472.CAN-04-0074 15172975

[52] Willett CG, Boucher Y, di Tomaso E, Duda DG, Munn LL, Tong RT, et al Direct evidence that the VEGF-specific antibody bevacizumab has antivascular effects in human rectal cancer. Nature Medicine. 2004;10(2):145–147. 10.1038/nm988 14745444 PMC2693485

[53] Batchelor TT, Gerstner ER, Emblem KE, Duda DG, Kalpathy-Cramer J, Snuderl M, et al Improved tumor oxygenation and survival in glioblastoma patients who show increased blood perfusion after cediranib and chemoradiation. Proceedings of the National Academy of Sciences. 2013;110(47):19059–19064. 10.1073/pnas.1318022110 PMC3839699 24190997

[54] Workman P, Aboagye EO, Balkwill F, Balmain A, Bruder G, Chaplin DJ, et al Guidelines for the welfare and use of animals in cancer research. British Journal of Cancer. 2010;102(11):1555–1577. 10.1038/sj.bjc.6605642 20502460 PMC2883160

[55] Pries AR, Secomb TW, Gaehtgens P, Gross JF. Blood flow in microvascular networks. Experiments and simulation. Circulation Research. 1990;67(4):826–834. 10.1161/01.RES.67.4.826 2208609

[56] Pries AR, Secomb TW, Gaehtgens P. Structure and hemodynamics of microvascular networks: heterogeneity and correlations. The American journal of physiology. 1995;269(5 Pt 2):H1713–22. 10.1152/ajpheart.1995.269.5.H1713 7503269

[57] Sweeney PW, Walker-Samuel S, Shipley RJ. Insights into cerebral haemodynamics and oxygenation utilising in vivo mural cell imaging and mathematical modelling. Scientific Reports. 2018;8(1):1373 10.1038/s41598-017-19086-z 29358701 PMC5778006

[58] Farid Z, Saleem AH, Al-Khazraji BK, Jackson DN, Goldman D. Estimating blood flow in skeletal muscle arteriolar trees reconstructed from in vivo data using the Fry approach. Microcirculation. 2017;24(5):e12378 10.1111/micc.12378 28470885

[59] Secomb TW. A Green’s function method for simulation of time-dependent solute transport and reaction in realistic microvascular geometries. Mathematical Medicine and Biology. 2016;33(4):475–494. 10.1093/imammb/dqv031 26443811 PMC5155623

[60] Shipley RJ, Smith AF, Sweeney PW, Pries AR, Secomb TW. A hybrid discrete–continuum approach for modelling microcirculatory blood flow. Mathematical Medicine and Biology: A Journal of the IMA. 2019;00(dqz006):1–18. 30892609 10.1093/imammb/dqz006

[61] Pries AR, Secomb TW. Microvascular blood viscosity in vivo and the endothelial surface layer. American journal of physiology Heart and circulatory physiology. 2005;289(6):H2657–H2664. 10.1152/ajpheart.00297.2005 16040719

[62] Pries AR, Ley K, Claassen M, Gaehtgens P. Red cell distribution at microvascular bifurcations. Microvascular research. 1989;38(1):81–101. 10.1016/0026-2862(89)90018-6 2761434

[63] Pries AR, Ley K, Gaehtgens P. Generalization of the Fahraeus principle for microvessel networks. The American journal of physiology. 1986;251(6 Pt 2):H1324–32. 10.1152/ajpheart.1986.251.6.H1324 3789184

[64] Pries AR, Secomb TW. Blood Flow in Microvascular Networks In: Comprehensive Physiology. Hoboken, NJ, USA: John Wiley & Sons, Inc; 2011 p. 3–36. Available from: http://doi.wiley.com/10.1002/cphy.cp020401.

[65] Baxter LT, Jain RK. Transport of fluid and macromolecules in tumors. II. Role of heterogeneous perfusion and lymphatics. Microvascular Research. 1990;40(2):246–263. 10.1016/0026-2862(90)90023-K 2250603

[66] Stylianopoulos T, Jain RK. Combining two strategies to improve perfusion and drug delivery in solid tumors. Proceedings of the National Academy of Sciences. 2013;110(46):18632–18637. 10.1073/pnas.1318415110 PMC3832007 24167277

[67] Sweeney PW, Walker-Samuel S, Shipley RJ. Vascular and interstitial flow solver for discrete microvascular networks. Zenodo. 2018.

[68] Secomb TW. Green’s function method for simulation of oxygen transport to tissue. http://physiologyarizonaedu/people/secomb/greens. 2011.

[69] Sanderson C, Curtin R. Armadillo: a template-based C++ library for linear algebra. Journal of Open Source Software. 2016;Vol. 1:p.26 10.21105/joss.00026

[70] Gaustad JV, Simonsen TG, Brurberg KG, Huuse EM, Rofstad EK. Blood supply in melanoma xenografts is governed by the morphology of the supplying arteries. Neoplasia (New York, NY). 2009;11(3):277–85. 10.1593/neo.81400 PMC2647730 19242609

[71] Morikawa S, Baluk P, Kaidoh T, Haskell A, Jain RK, McDonald DM. Abnormalities in pericytes on blood vessels and endothelial sprouts in tumors. The American journal of pathology. 2002;160(3):985–1000. 10.1016/S0002-9440(10)64920-6 11891196 PMC1867175

[72] Curry F. Mechanics and thermodynamics of transcapillary exchange, Handbook of Physiology, Section 2: The Cardiovascular System; 1984.

[73] Brace RA, Guyton AC. Transmission of Applied Pressure Through Tissues: Interstitial Fluid Pressure, Solid Tissue Pressure, and Total Tissue Pressure. Experimental Biology and Medicine. 1977;154(2):164–167. 10.3181/00379727-154-39628 840854

[74] Lee CG, Heijn M, Di Tomaso E, Griffon-Etienne G, Ancukiewicz M, Koike C, et al Anti-vascular endothelial growth factor treatment augments tumor radiation response under normoxic or hypoxic conditions. Cancer Research. 2000;60(19):5565–5570. 11034104

[75] Soltani M, Chen P. Numerical modeling of fluid flow in solid tumors. PLoS ONE. 2011;6(6):e20344 10.1371/journal.pone.0020344 21673952 PMC3108959

[76] Kim E, Stamatelos S, Cebulla J, Bhujwalla ZM, Popel AS, Pathak AP. Multiscale imaging and computational modeling of blood flow in the tumor vasculature. Annals of Biomedical Engineering. 2012;40(11):2425–2441. 10.1007/s10439-012-0585-5 22565817 PMC3809908

[77] El-Emir E, Boxer GM, Petrie IA, Boden RW, Dearling JLJ, Begent RHJ, et al Tumour parameters affected by combretastatin A-4 phosphate therapy in a human colorectal xenograft model in nude mice. European Journal of Cancer. 2005;41(5):799–806. 10.1016/j.ejca.2005.01.001 15763657

[78] Simonsen TG, Gaustad JV, Leinaas MN, Rofstad EK. High Interstitial Fluid Pressure Is Associated with Tumor-Line Specific Vascular Abnormalities in Human Melanoma Xenografts. PLoS ONE. 2012;7(6):e40006 10.1371/journal.pone.0040006 22768196 PMC3386940

[79] Yuan F, Dellian M, Fukumura D, Leunig M, Berk DA, Torchilin VP, et al Vascular permeability in a human tumor xenograft: molecular size dependence and cutoff size. Cancer research. 1995;55(17):3752–6. 7641188

[80] Yuan F, Chen Y, Dellian M, Safabakhsh N, Jain RK. Time-dependent vascular regression and permeability changes in established human tumor xenografts induced by an anti-vascular endothelial growth factor/vascular permeability factor antibody. Proceedings of the National Academy of Sciences. 1996;93(25):14765–14770. 10.1073/pnas.93.25.14765 PMC26210 8962129

[81] Chauhan VP, Stylianopoulos T, Martin JD, PopoviÄ Z, Chen O, Kamoun WS, et al Normalization of tumour blood vessels improves the delivery of nanomedicines in a size-dependent manner. Nature Nanotechnology. 2012;7(6):383–388. 10.1038/nnano.2012.45 22484912 PMC3370066

[82] Hernández-Agudo E, Mondejar T, Soto-Montenegro ML, Megías D, Mouron S, Sanchez J, et al Monitoring vascular normalization induced by antiangiogenic treatment with 18F-fluoromisonidazole-PET. Molecular Oncology. 2016;10(5):704–718. 10.1016/j.molonc.2015.12.011 26778791 PMC5423153

[83] Ballard K, Perl W. Osmotic reflection coefficients of canine subcutaneous adipose tissue endothelium. Microvascular Research. 1978;16(2):224–236. 10.1016/0026-2862(78)90057-2 739904

[84] Jain RK, Tong RT, Munn LL. Effect of vascular normalization by antiangiogenic therapy on interstitial hypertension, peritumor edema, and lymphatic metastasis: Insights from a mathematical model. Cancer Research. 2007;67(6):2729–2735. 10.1158/0008-5472.CAN-06-4102 17363594 PMC3022341

[85] Baluk P, Hashizume H, McDonald DM. Cellular abnormalities of blood vessels as targets in cancer. Current Opinion in Genetics and Development. 2005;15(1):102–111. 10.1016/j.gde.2004.12.005 15661540

[86] Gullino PM, Swabb EA, Wei J, Gullino PM. Diffusion and Convection in Normal and Neoplastic Tissues. Cancer Research. 1974;34(10):2814–2822. 4369924

[87] Nia HT, Liu H, Seano G, Datta M, Jones D, Rahbari N, et al Solid stress and elastic energy as measures of tumour mechanopathology. Nature Biomedical Engineering. 2017;1(1). 10.1038/s41551-016-0004 PMC5621647 28966873

