# Supplementary figures and images for: Modelling the transport of fluid through heterogeneous, whole tumours in silico
Source: PLoS Comput Biol. 2019 Jun 21;15(6):e1006751. doi: 10.1371/journal.pcbi.1006751 (PMC6588205; doi:10.1371/journal.pcbi.1006751)

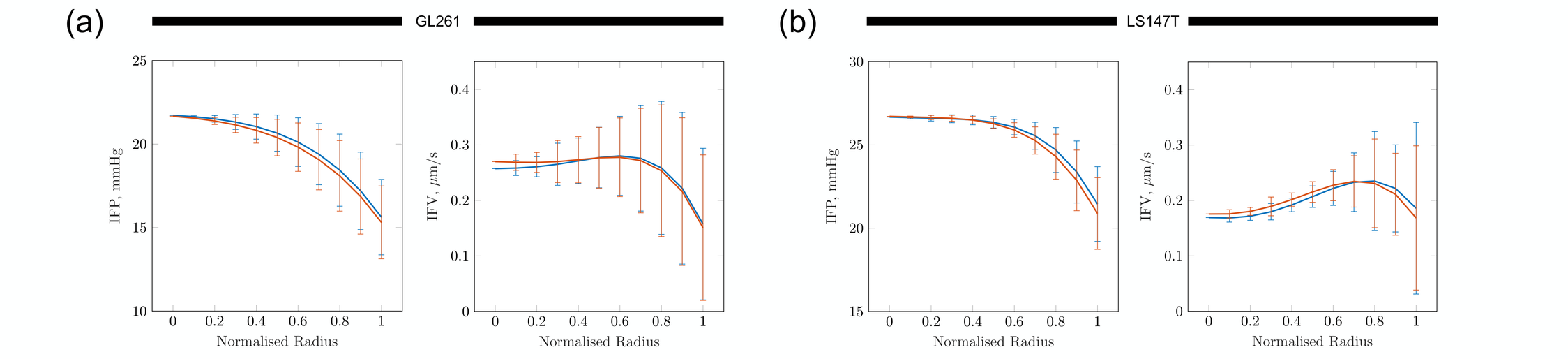

Supplement: S1 Fig — Plots show (left) IFP and (right) IFV for (blue) pre- and (red) post-normalization. Error bars represent standard deviation. (TIF) [file pcbi.1006751.s001.tif]

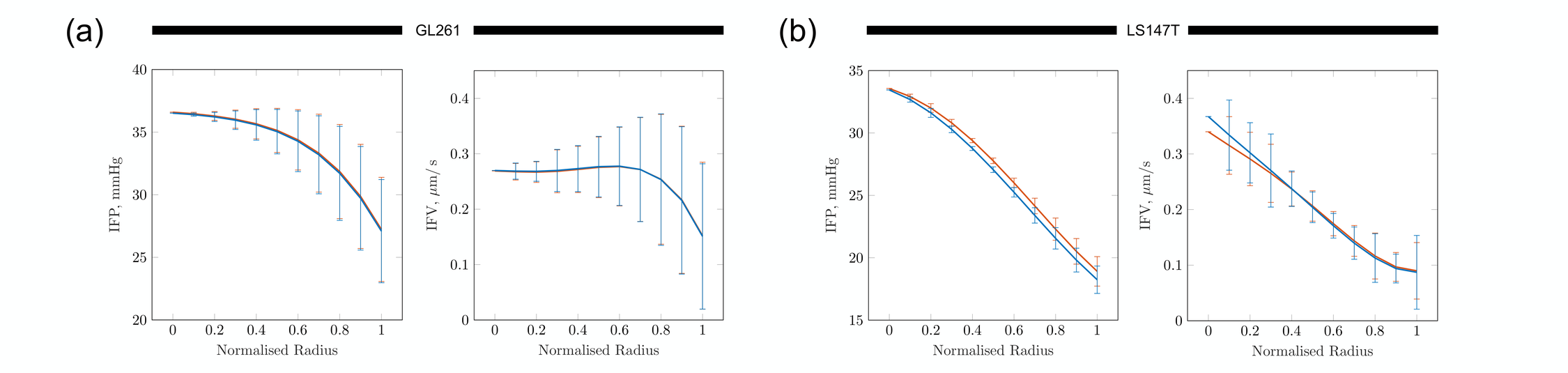

Supplement: S2 Fig — Plots show (left) IFP and (right) IFV for normalization of (blue) Lp, σ, vessel diameters and κ, and (red) κ. Error bars represent standard deviation. (TIF) [file pcbi.1006751.s002.tif]

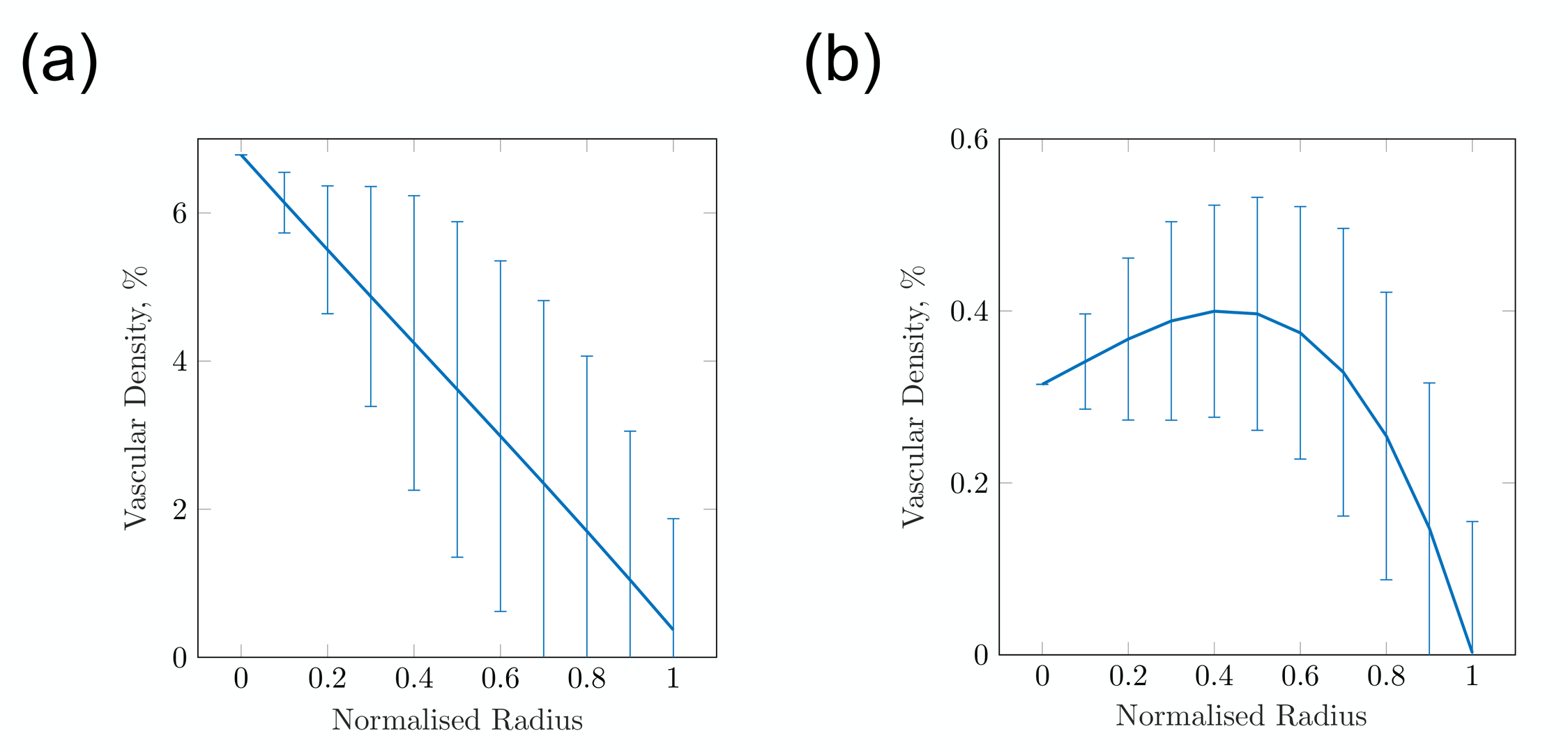

Supplement: S3 Fig — Error bars represent standard deviation. (TIF) [file pcbi.1006751.s003.tif]
